# Cancer Markers Selection Using Network-Based Cox Regression: A Methodological and Computational Practice

**DOI:** 10.3389/fphys.2016.00208

**Published:** 2016-06-17

**Authors:** Antonella Iuliano, Annalisa Occhipinti, Claudia Angelini, Italia De Feis, Pietro Lió

**Affiliations:** ^1^Istituto per le Applicazioni del Calcolo “Mauro Picone,” Consiglio Nazionale delle RicercheNaples, Italy; ^2^Computer Laboratory, University of CambridgeCambridge, UK

**Keywords:** cancer, Cox model, high-dimensionality, gene expression, network, regularization, survival

## Abstract

International initiatives such as the Cancer Genome Atlas (TCGA) and the International Cancer Genome Consortium (ICGC) are collecting multiple datasets at different genome-scales with the aim of identifying novel cancer biomarkers and predicting survival of patients. To analyze such data, several statistical methods have been applied, among them Cox regression models. Although these models provide a good statistical framework to analyze omic data, there is still a lack of studies that illustrate advantages and drawbacks in integrating biological information and selecting groups of biomarkers. In fact, classical Cox regression algorithms focus on the selection of a single biomarker, without taking into account the strong correlation between genes. Even though network-based Cox regression algorithms overcome such drawbacks, such network-based approaches are less widely used within the life science community. In this article, we aim to provide a clear methodological framework on the use of such approaches in order to turn cancer research results into clinical applications. Therefore, we first discuss the rationale and the practical usage of three recently proposed network-based Cox regression algorithms (i.e., Net-Cox, AdaLnet, and fastcox). Then, we show how to combine existing biological knowledge and available data with such algorithms to identify networks of cancer biomarkers and to estimate survival of patients. Finally, we describe in detail a new permutation-based approach to better validate the significance of the selection in terms of cancer gene signatures and pathway/networks identification. We illustrate the proposed methodology by means of both simulations and real case studies. Overall, the aim of our work is two-fold. Firstly, to show how network-based Cox regression models can be used to integrate biological knowledge (e.g., multi-omics data) for the analysis of survival data. Secondly, to provide a clear methodological and computational approach for investigating cancers regulatory networks.

## Introduction

Recent developments in high-throughput technology have produced a huge amount of multiple and diverse genome-scale data to deal with biological and clinical questions in cancer. For example, genomics, transcriptomics, and epigenomics information is nowadays publicly available for tens of different cancer cell lines from thousands of patients in The Cancer Genome Atlas (TCGA, http://cancergenome.nih.gov/). Mutations data over one million tumor samples are also reported in Cosmic (http://cancer.sanger.ac.uk/cosmic), the world's largest and most comprehensive resource for exploring the impact of somatic mutations. Other valuable databases include The Gene Expression Omnibus (GEO, http://www.ncbi.nlm.nih.gov/gds) among others. Such amount of data is likely to revolutionize genetics and biomedical cancer research, but a thorough integration of all these different types of information is necessary. Indeed, cancer is a “multi-factorial” disease caused by a combination of genetic, environmental, and lifestyle factors. Such factors play an important role in discovering prognostic and diagnostic cancer gene signatures opening a new way toward the so called “personalized medicine.” The term refers to a new type of therapy that is essentially based on the features of each patient. For instance, the anticancer drug Cetuximab (Karapetis et al., 2008) inhibits cells proliferation by binding to the EGF receptor and, consequently, preventing activation of the downstream signaling pathway. However, it has been found that Cetuximab can work only if the K-RAS gene is not mutated. Another example is the anti-cancer drug Trastuzumab (Hudis, 2007), which is effective only in patients that highly express the human epidermal growth factor (HER2) at the cell surface, to which the antibody binds. These examples highlight the need of identifying stable and interpretable biomarkers able to predict patient survival and characterize a patient-personalized therapy. In addition, the knowledge of complex cancer processes and networks is important to optimize the use of technology within health care (Raghupathi and Raghupathi, 2014). By discovering associations within the data, big data analytics has the potential to improve care, save lives, and lower costs.

As a consequence, in the last years, there has been a growing interest in developing methods that integrate different genome-scale data into regression models for survival data to create a comprehensive view of human biology and disease (Wang et al., 2014). A popular used approach for the integration of genomic and clinical information is the Cox proportional hazard model (Cox, 1972). The main goal of such method is investigating the connection between gene expression data and survival information to predict cancer survival, assess cancer outcomes, and identify new gene markers. However, since gene expression data are usually characterized by a number of covariates *p* much larger than the sample size *n*, the traditional Cox model cannot be applied. Hence, several penalized Cox regression methods have been developed to identify core pathways and biomarkers involved in cancer progression, e.g., the Cox model based on Lasso penalty (Tibshirani, 1996, 1997; Gui and Li, 2005). Alternative penalized Cox regression models based on variable selection include the SCAD (Fan and Li, 2001), the adaptive Lasso (Zou, 2006), the elastic net model (Zou and Hastie, 2005; Simon et al., 2011a; Wu, 2012), and the Dantzig selector (Candes and Tao, 2007) among others. These methods are able to cope with the high-dimensionality of gene expression data, thus solving the “*p* ≫ *n*” issue (Engler and Li, 2009). All these penalized models are statistically efficient in high-dimensional regression, but they perform poorly on data with high collinearity. Moreover, no biological knowledge is taken into account. Indeed, they are simply based on statistical frameworks completely ignoring biological regulatory network, protein–protein interaction (PPI), signaling pathways, and well-known relationships among genes. In such models, the lack of biological information produces instability in predictors reducing the predictive ability of the models. Hence, in order to provide more reliable and biologically meaningful results, the inclusion of *a-priori* biological knowledge into the models is mandatory. To address this issue, new penalized Cox methods based on the integration of genomic information have been recently proposed (Zhang et al., 2013; Gong et al., 2014; Sun et al., 2014). In such models, the genomic information is encoded by a network whose graph structure identifies a given relation (edges) between genes (nodes). The resulting Laplacian matrix is then integrated as penalty in the Cox regression models. In particular, the network can represent the correlation between genes (Zhang et al., 2013), KEGG pathways identification (Sun et al., 2014), functional interaction network (Huttenhower et al., 2009), or PPI. These Cox models based on *a-priori* biological network are called “network-based Cox regression.”

The network-based Cox regression methods provide an efficient tool to perform Cox regression on high-dimensional data incorporating genes network information. In literature, there are some recent approaches that analyze different Cox methods. For instance, an accurate review of eight different methods that integrate network information into multi-variable Cox models is presented to study the risk prediction in breast cancer and the integrated Brier score is used as a performance measure (Fröhlich, 2014). However, the study performed enrichment analysis on the signatures genes selected by the compared models without showing any survival prediction analysis in terms of Kaplan–Meier curves. A network-based Cox regression model that explores gene-to-gene connections in multiple cancer datasets is also performed for maximizing the overall association of the sub-network with clinical outcomes (Martinez-Ledesma et al., 2015). A potential limitation of these conventional networks is that the edges only reflect the information of within-features or within-relations, and do not consider the association between features and outcomes, which may be useful in improving the predictive power. Therefore, an alternative network construction method for the outcome-guided gene-interaction network has to be introduced in order to improve the performance of survival analysis in network-based Cox regression (Jeong et al., 2015).

**Figure 1:**
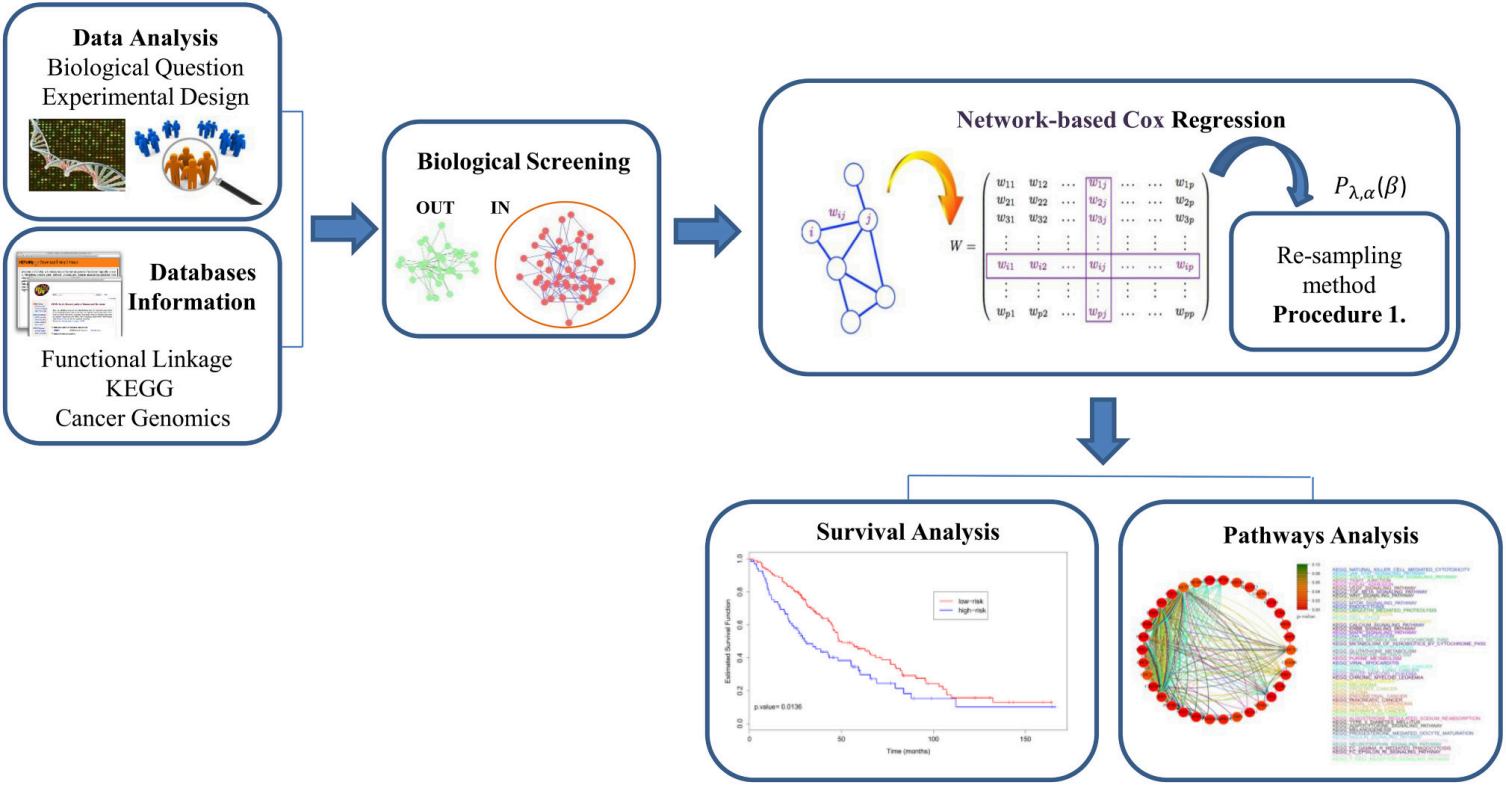
**The pipeline of network-based Cox models approach for cancer survival analysis in four general steps**. (1) Define the biological question and the experimental design and then, integrate *a-priori* biological information using functional map of the human genome; (2) perform biological screening of the data in order to select IN variables to use in the analysis; (3) implement network-based Cox regression models with the integration of a re-sampling method based on a cross-validated approach; (4) apply survival analysis to predict cancer patients and pathway analysis to explore groups of genes associated to the disease.

In this work, we present a methodological framework for the analysis of molecular and survival data through a cross-validated approach of network-based Cox regression algorithms (*Net-Cox, Adalnet*, and *fastcox*, see Section Methods). The method starts from the analysis of raw data and, through a cross-validated penalty approach, it guides the reader to the interpretation of the final results. As shown in Figure 1, the general steps of our approach are the following: (i) defining the biological question and the experimental design using microarray data, then integrating *a-priori* biological information using functional map of the human genome such as HEFalMp (Huttenhower et al., 2009) and KEGG; (ii) performing biological screening of the data for selecting relevant features through cross-validated penalization (Simon et al., 2011b); (iii) implementing network-based Cox regression models for the analysis of cancer-related genes; (iv) evaluating survival models to predict cancer patient prognosis and exploring cancer associated pathways. The presented approach provides a new methodological framework for the study and the interpretation of regression methods through gene-network and pathways analyses and it can be easily adapted to incorporate other network-based Cox regression algorithms.

**Figure 2:**
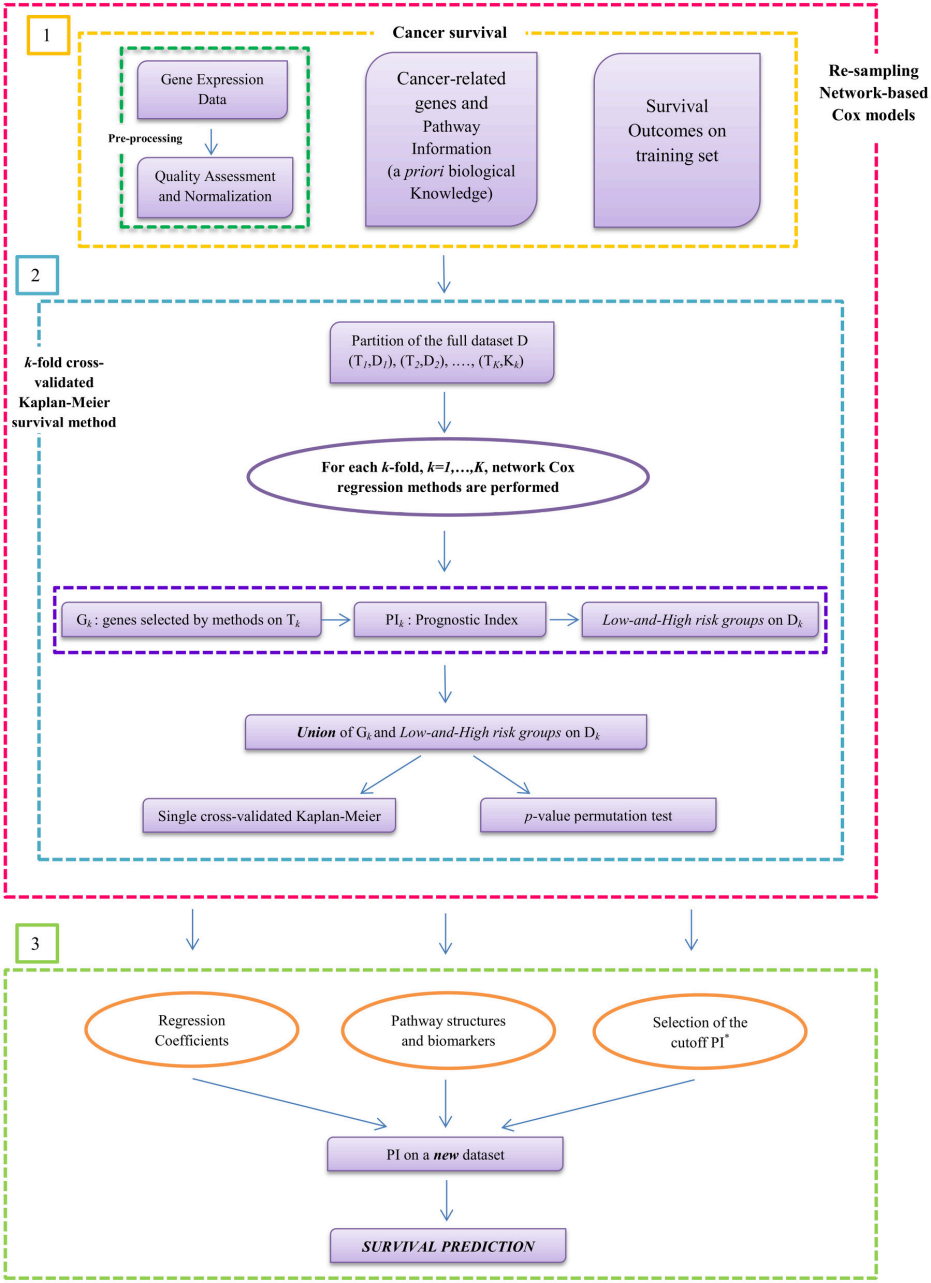
**Workflow of prognostic model building by using gene expression profile in cancer**. The method starts from the analysis of raw data and, through a cross-validated penalty approach, it leads to the interpretation of the final results. Step (1) includes the input data for the survival analysis: gene expression data, cancer-related genes, pathway information, and overall survival (OS) times. Step (2) illustrates the novelty of the work based on a k-fold cross-validation Kaplan–Meier procedure by integrating network-regularized Cox models for selecting significant genes and pathways structures. The Prognostic Index (*PI*) has been used to divide the patients in high-risk and low-risk groups. Then, the union of these two groups is done to plot single cross-validated Kaplan–Meier curves and to calculate the *p*-value permutation test. Step (3) shows the survival prediction to test how well the models generalize across independent cancer datasets.

A preliminary study for the comparison of penalized Cox models was presented in Iuliano et al. (2014), where the analysis was limited to cancer survival prediction using top ranked genes. No simulation studies, extensive pathways analysis or validation of the data were performed in that study. On the contrary, this article presents a more accurate and complete analysis based on a cross-validated approach (Simon et al., 2011b), the overall workflow (see Figure 2) that includes both simulation studies and novel real cancer datasets (see Section Data Analysis). Simulated data have been used to perform a statistical comparison of the methods in terms of sensitivity, specificity, number of selected genes, false positive rates, and Matthews correlation coefficient in two simulation settings with different genetic effects. On the other hand, real datasets analysis was performed to assess the relevance of the selected genes in the training dataset and to test the survival prediction accuracy of each model. Cross-validated Kaplan–Meier curves for survival analysis and pathway analysis were also computed (see Section Results). The novelty of the current study consists in the integration of a cross-validated approach (Simon et al., 2011b) to obtain an accurate survival prediction even when the number of cases is relatively small for an effective sample splitting (see Figure 2). Cross-validation methods have been largely applied in Cox regression models to estimate prediction errors and for model parameters tuning (Vasselli et al., 2003; Molinaro et al., 2005; Simon et al., 2011b). Some of the most relevant cross-validation approaches include leave-one-out cross-validation (LOOCV; Kearns and Ron, 1999), *k*-fold (Refaeilzadeh et al., 2009), and bootstrap algorithms (Kohavi, 1995). However, all these methods do not provide a good estimation if the data available are limited for an effective division in training and test sets. On the contrary, the cross-validation method used in our analysis (Simon et al., 2011b) is based on a re-sampling algorithm that allows an accurate prediction of the survival risk model regardless the data size. Therefore, in this work, we first present a novel statistical approach to infer pathway interaction networks from gene expression data that relies on a new mathematical concept (based on the biological screening and network-based Cox regression methods) for understanding pathways' activity and relationships. Second, we provide a methodological strategy to researchers for the use of network-based Cox regression models in order to turn cancer research results into clinical applications.

## Methods

### Network-regularized Cox regression models

The Cox Proportional hazards model (Cox, 1972) is the most widely used model to describe the relationship between survival times and predictor covariates.

Given a sample of *n* subjects, let *T*_*i*_ and *C*_*i*_ be the survival time and the censoring time, respectively, for subject *i* = 1, …, *n*. Let *t*_*i*_ = min {*T*_*i*_, *C*_*i*_} be the observed survival time and δ_*i*_ = *I*(*T*_*i*_ ≤ *C*_*i*_) the censoring indicator, where *I*(·) is the indicator function (i.e., δ_*i*_ = 1 if the survival time is observed and δ_*i*_ = 0 if the survival time is censored). We denote by $X_{i}=(X_{i1},\ldots ,X_{ip}{)}^{\prime }$ the regression vector of *p*-variables for the *i*th subject (i.e., the gene expression profile of the *i*th patient over *p* genes). The survival time *T*_*i*_ and the censoring time *C*_*i*_ are assumed to be conditionally independent given ***X***_*i*_. Furthermore, the censoring mechanism is assumed to be non-informative. The observed data can be represented by the triplets {(*t*_*i*_, δ_*i*_, ***X***_*i*_), *i* = 1, …, *n*}. The Cox regression method assumes that the hazard function *h*(*t*|**X**_*i*_), which is the risk of death at time *t* for the *i*th patient with gene expression profile ***X***_*i*_, can be written as

$$h(t|X_{i})=h_{0}(t)\exp\left(X_{i}^{\prime }\beta \right)$$

where *h*_0_(*t*) is the baseline hazard and $\beta =(\beta_{1},\ldots ,\beta_{p}{)}^{\prime }$ is the column vector of the regression parameters.

In the classical setting, the regression coefficients are estimated by maximizing the Cox's log-partial likelihood

(1) $$pl(\beta )=\sum_{i=1}^{n}\delta_{i}\left\{X_{i}^{\prime }\beta -\log\left[\sum_{j\in R(t_{i})}\exp(X_{j}^{\prime }\beta )\right]\right\},$$

where *t*_*i*_ is the survival time (observed or censored) for the *i*th patient, *R*(*t*_*i*_) is the risk set at time *t*_*i*_ (i.e., the set of all patients who still survived prior to time *t*_*i*_).

However, in the analysis of gene expression data, the number of genes *p* is usually larger than the sample size *n* and the standard Cox-model cannot be directly applied. To cope with the curse of dimensionality (*p* ≫ *n*), a variety of penalization approaches have been proposed for achieving good prediction performance and easy interpretation of the data. Although these regularization methods induce sparsity into the solution by shrinking some estimates to zero, the biological relationship of gene expression profiles is not taken into account. Hence, in order to integrate information from molecular interactions between genes, network-based constrained methods for high-dimensional Cox regression have been introduced.

In this context, the regression coefficients are estimated by maximizing the penalized Cox's log-partial likelihood function

(2) $$pl_{pen}(\beta )=\sum_{i=1}^{n}\delta_{i}\left\{X_{i}^{\prime }\beta -\log\left[\sum_{j\in R(t_{i})}\exp(X_{j}^{\prime }\beta )\right]\right\}-P_{\lambda }(\beta ),$$

where *P*_λ_(***β***) is a network-constrained penalty function on the coefficients ***β***.

Such penalty function describes the existing relationships among the covariates (genes) specified by a network *G* = (*V, E, W*) (weighted and undirected graph), where *V* = {1, …, *p*} is the set of vertices (genes/covariates), an element (*i, j*) in the edge set *E* ⊂ *V* × *V* indicates a link between vertices *i* and *j* and *W* = (*w*_*ij*_), (*i, j*) ∈ *E* is the set of weights associated with the edges. These weights are usually used to represent the relations between genes in terms of gene–gene interaction, KEGG pathway analysis or PPI. Hence, the network structure plays an important role since it incorporates prior gene regulatory information often ignored.

The three regularized network-based Cox regression models used in our study are presented below and differ in the form of the penalty function *P*_λ_(***β***).

#### Net-Cox method

Net-Cox regression (Zhang et al., 2013) is an extension of the *L*_2_-Cox model and uses the following penalty function

(3) $$P_{\lambda ,\alpha }(\beta )=\lambda \left[\alpha \|\beta {\|}_{2}^{2}+(1-\alpha )\Phi (\beta )\right],$$

where λ > 0 and α ∈ (0, 1] are two regularization parameters in the network constraint. and

(4) $$\Phi (\beta )=\sum_{(i,j)\in E}w_{ij}{\left(\beta_{i}-\beta_{j}\right)}^{2}.$$

The penalty (3) consists of two terms: the first one is an *L*_2_-norm of ***β*** that regularizes the uncertainty in the network constraint; the second term is a network Laplacian penalty Φ(***β***) that encourages smoothness among correlated gene in the network and encode prior knowledge from a network.

Given a normalized graph weight matrix *W*, we assume that co-expressed (related) genes are assigned similar coefficients by defining the cost term Φ(***β***) as reported in Equation (4). Φ(***β***) can be also written as $\Phi (\beta )=\beta^{\prime }(\text{I}-\text{W})\beta =\beta^{\prime }\bar{L}\beta$ where L is a positive semi-definite matrix derived from network information (weight matrix **W**) and **I** is an identity matrix. Hence, the objective function will result in a significant cost in the network if any pair of genes is connected by an high weight edge and the difference between their coefficients is large.

Note that to identify the signature genes classified by *Net-Cox*, which is a ridge regression based method, we create a consensus ranking of the relevant cancer genes.

#### AdaLnet method

*Ada*ptive *L*aplacian *net* (Sun et al., 2014) is a modified version of a network-constrained regularization procedure for fitting linear models and for variable selection (Li and Li, 2008, 2010) where the predictors are genomic data with graphical structures. *AdaLnet* is based on prior gene regulatory network information, represented by an undirected graph for the analysis of gene expression data and survival outcomes.

Denoting with $d_{i}=\underset{i:\left(i,j\right)\in E}{\sum }w_{ij}$ the degree of vertex *i*, *AdaLnet* defines the normalized Laplacian matrix **L** = (*l*_*ij*_) of the graph G by

(5) $$l_{i,j}=\;\{\begin{array}{cc} \;1, & \text{ if}i=j\text{ and}d_{i}\neq 0,\\ -w_{ij}/\sqrt{d_{i}d_{j}}, & \text{ if}(i,j)\in E,\\ \;0, & \text{ otherwise}. \end{array}$$

Note that **L** is positive semi definite. The network-constrained penalty in Equation (2) is given by

(6) $$P_{\lambda ,\alpha }(\beta )=\lambda [\alpha \|\beta {\|}_{1}+(1-\alpha )\Psi (\beta )],$$

with

(7) $$\Psi (\beta )=\sum_{(i,j)\in E}w_{ij}{\left(\text{sign}({\tilde{\beta }}_{i})\beta_{i}/\sqrt{d_{i}}-\text{sign}({\tilde{\beta }}_{j})\beta_{j}/\sqrt{d_{j}}\right)}^{2}.$$

Equation (6) is composed by two penalty terms. The first one is an *L*_1_-penalty that induces a sparse solution, the second one is a quadratic Laplacian penalty $\Psi (\beta )=\beta^{\prime }\tilde{L}\beta$ that imposes smoothness of the parameters ***β*** between neighboring vertices in the network. Note that $\tilde{L}=S^{\prime }LS$ with $S=\text{diag}(\text{sign}({\tilde{\beta }}_{1}),\ldots ,\text{sign}({\tilde{\beta }}_{p}))$ and $\tilde{\beta }=({\tilde{\beta }}_{1},\ldots ,{\tilde{\beta }}_{p})$ is obtained from a preliminary regression analysis. The scaling of the coefficients ***β*** respect to the degree allows the genes with more connections (i.e., the hub genes) to have larger coefficients. Hence, small changes of expression levels of these genes can lead to large changes in the response.

An advantage of using penalty (7) consists in representing the case when two neighboring variables have opposite regression coefficient signs, which is reasonable in network-based analysis of gene expression data. Indeed, when a transcription factor (TF) positively regulate gene *i* and negatively regulate gene *j* in a certain pathway, the corresponding coefficients will result with opposite sign.

Note that in *Net-Cox* and *AdaLnet*, λ is the parameter controlling the weight between the likelihood and the network constraint and α ∈ (0, 1] is the parameter weighting the network constraint.

#### Fastcox method

The penalty function of *fastcox* (Yang and Zou, 2012) computes the solution paths of the elastic net penalized Cox's proportional hazards model (Wu, 2012). In this method the penalty function in Equation (2) is given by

$$P_{\lambda ,\alpha }(\beta )=\lambda \left[\alpha w\|\beta {\|}_{1}+\frac{1}{2}(1-\alpha )\|\beta {\|}_{2}^{2}\right],$$

where the non-negative weights *w* allow a more flexible estimation. In particular, setting *w*_*j*_ = 0 implies no shrinkage and the variable *j* will be always included in the final model. Default is 1 for all variables. α ∈ (0, 1] is the elastic net trade off. This regularization technique is a combination of the lasso and ridge penalty that produce a sparse model (given by the *L*_1_-penalty) with good prediction accuracy, while encouraging a grouping effect. It is worthy to note that this method does not include any gene network information. It has been used in our study to obtain pathways investigation and survival prediction from a relevant method that is simply based on statistical framework.

### Tuning parameters by five-fold cross-validation

For all the methods, we estimated the regularization parameters using cross-validation. Four-folds of data are used to build a model for validation on the fifth fold, cycling through each of the five-folds in turn. Then, the (λ,α) pair that minimizes the cross-validation log-partial likelihood (CVPL) are chosen as the optimal parameters. CVPL is defined as

(8) $$CVPL(\lambda ,\alpha )=-\frac{1}{n}\sum_{k=1}^{K}\left\{\ell ({\hat{\beta }}^{\left(-k\right)}(\lambda ,\alpha ))-\ell^{\left(-k\right)}({\hat{\beta }}^{\left(-k\right)}(\lambda ,\alpha ))\right\},$$

where ${\hat{\beta }}^{\left(-k\right)}\left(\cdot \right)$ is the estimate obtained from excluding the *k*th part of the data with a given pair of (λ, α), ℓ(·) is the Cox log-partial likelihood on all the sample and ℓ^(−*k*)^(·) is the log-partial likelihood when the *k*th fold is left out (van Houwelingen et al., 2006).

### General algorithm: A re-sampling method for survival prediction

The prediction capabilities of a given method are usually evaluated using a training set to select the markers and a testing set to measure the goodness of the prediction. In several cases training and test sets are obtained splitting a given dataset in two parts. However, findings could be over optimistic depending on the specific split. To further understand the role of the network information in cross-validation and to overcome the drawbacks of investigating only one split, each network-based model was validated with the re-sampling procedure suggested by Simon et al. (2011b). This method is based on a cross-validated estimate of the survival distribution of the risk groups and provide a more efficient use of data than fixed sample splitting (see Figure 2). The steps of the re-sampling algorithm for survival prediction are presented below.

**Procedure 1**: *k*-fold Cross-validated Kaplan–Meier survival method

1. The full dataset *D* is partitioned into *K* approximately equal parts *D*_1_, …, *D*_*K*_.
  For each *k* = 1, …, *K*
2. Set *T*_*k*_ = *D* − *D*_*k*_ as the training set and *D*_*k*_ as the testing set.
3. Perform network-based Cox regression on *T*_*k*_ and select high-risk cancer genes *G*_*k*_. Denote the parameter estimate by ${\hat{\beta }}_{T_{k}}$.
4. Calculate the prognostic index (*PI*) for each patient *i*_*k*_ in *D*_*k*_ as
  $$PI_{i_{k}}^{D_{k}}=x_{i_{k}}^{\prime }{\hat{\beta }}_{T_{k}},$$
  where *x*_*i*_*k*__ is the vector of gene expression value associated to the *i*_*k*_-th patient into the *k*-fold. Each patient *i*_*k*_ in *D*_*k*_ is assigned into the *high/low-risk* group if its prognostic index $PI_{i_{k}}^{D_{k}}$ is above (or below) a fixed threshold $PI^{*,T_{k}}$ defined adaptively on *T*_*k*_.
5. All the patients classified as *low-and-high risk* in any of the folds are grouped together and a single Kaplan–Meier curve is computed as the union of the risk groups defined in each fold. The set of predictive genes is selected as the union of *G*_*k*_, for *k* = 1, …, *K*.
6. Compute the log-rank $\chi_{0}^{2}$ statistic under the null hypothesis that survival is independent of expression profile.
7. Calculate a permutation *p*-value as follows:
  - from the *m*-th permutation data (*m* = 1, …, *M*), compute the log-rank $\chi_{b}^{2}$ statistic using the cross-validation procedure (1–6),
  - compute the permutation *p*-value, $\hat{p}$, as
    $$\hat{p}=M^{-1}\sum_{i=1}^{M}I(P_{m}\geq P_{0}).$$

For our analysis, the estimate ${\hat{\beta }}_{T_{k}}$ in step 4 was computed by using five-fold cross-validation (i.e., *K* = 5) to select the optimal tuning parameter values (${\hat{\lambda }}_{T_{k}},{\hat{\alpha }}_{T_{k}}$), that we used to fit the corresponding penalized function $P_{{\hat{\lambda }}_{T_{k}},{\hat{\alpha }}_{T_{k}}}\left({\hat{\beta }}_{T_{k}}\right)$ on *T*_*k*_. In particular, we first set α to a sufficiently fine grid of values on [0, 1]. For each fixed α, λ was chosen from {10^−5^, 10^−4^, 10^−3^, 10^−2^, 10^−1^, 1} for *Net-Cox*, while it was set λ to a decreasing sequence of values λ_*max*_ to λ_*min*_ automatically chosen for *AdaLnet* and *fastcox*.

In step 5, we selected $PI^{*,T_{k}}$ as the optimal cut-off in terms of $PI^{D_{k}}$. By using the $PI_{i_{k}}^{T_{k}}$, it was possible to split the patients in two subgroups, i.e., *high-risk* and *low-risk* prognosis groups. Thus, the patient *i*_*k*_ in *T*_*k*_ was assigned to the *high-risk* (or *low-risk*) group if his prognostic index $PI_{i_{k}}^{T_{k}}$ was above (or below) the quantile selected on a grid of given values that spans from 30 to 70%. The cut-off $PI^{*,T_{k}}$ was chosen in correspondence to the lowest *p*-value in a log rank test on this grid.

In step 7, we set *M* equal to 500.

### Survival analysis

Network-based Cox regression model was used to discover significant variables, i.e., genes, correlated with death risk. Overall survival (OS) curves were estimated using the Cross Kaplan–Meier estimator and compared using the two-sided log-rank test as implemented in the R package *survival*. The statistical significance of the log-rank statistic related to the cross-validated Kaplan–Meier curves was obtained through a permutation distribution (Simon et al., 2011b) as described in the previous section. Permutation test was used to test the association between *high-risk* or *low-risk groups* and *p* < 0.05 were considered statistically significant. A simple scheme of the applied procedure for OS estimation is reported in Figure 2.

Furthermore, we also validated the predictive performance of the three methods using independent dataset for training and testing. In this context, we used the largest dataset as training set to identify the gene expression signatures (see Figure 2, step 2). Then, the second independent dataset was considered as test set in order to analyze the survival prediction of the models. We used Kaplan–Meier survival curves and log-rank test to perform the analysis (see Figure 2, step 3).

### Pathway analysis

We performed pathway analysis based on KEGG database and on the Human Experimental/Functional Mapper (Huttenhower et al., 2009). In particular, we focused on a gene–gene interaction analysis developing gene-networks that describe the relations between genes in terms of KEGG pathways. Each node in the network represents a gene and an edge between two nodes means that the two genes belongs to the same pathway. Different colors are used for different pathways. The color of each node indicates how strong is the relationship between the gene and the disease under analysis (ovarian and breast cancer; Huttenhower et al., 2009). The *p*-value chosen within the interval [0, 0.1] represents the node color intensity. Red color, that is *p* = 0, means that there is a high significant gene-disease relation, while green color, that is *p* = 0.1, means that not exist a relevant gene-disease relation.

Gene networks have been computed by considering only the *not isolated* genes in the intersection between KEGG pathways and the set of genes selected by each method. Given a set of genes *G* and the set of all the KEGG pathways *K*, we defined a gene *g* as *not isolated* if *G* ∩ *K* ⊉ {*g*}. Namely, *g* is *not isolated* if there is at least another gene *g*′ ∈ *G* belonging to the same pathways of *g*.

### Software

The methodological approach presented in Figure 2 has been implemented as an integrative R script that allows to run the different algorithms under the same R environment. *Net-Cox*, which is a Matlab toolbox (http://compbio.cs.umn.edu/Net-Cox/), *AdaLnet*, available as an R code and sent us upon request and *fastcox*, which is an R package (http://code.google.com/p/fastcox/) were merged together by using *R.matlab*, https://cran.r-project.org/web/packages/R.matlab/index.html. The script also includes the implementation of the re-sampling permutation approach (Simon et al., 2011b) and the cross-validation method for parameters estimation. Both simulated and real data can be used to run the script which can be easily adapted for the integration of new Cox models.

For real data analysis, the microarray data were preprocessed using R packages available in Bioconductor. First, we selected from the initial dataset the genes that were more likely to be involved in cancer by using a functional map summarizing the most relevant interactions in the cancer area of interest (Huttenhower et al., 2009). Then, we used HEFaIMp tool (Huttenhower et al., 2009) to build the genes network and identify the weight of the edges between the selected genes. Finally, *Net-Cox, AdaLnet*, and *fastcox* were implemented integrating a cross-validation method for selecting the optimal tuning parameters λ and α and a re-sampling based procedure (Simon et al., 2011b), see Procedure 1.

The scripts are available upon request from the first two authors.

## Data analysis

### Simulation scheme

We used the three methods in two different simulation settings (Wu and Wang, 2013; Sun et al., 2014) in order to investigate the performances and the properties of the three models and to facilitate the interpretation of results. We considered two scenarios that are likely to be encountered in genomic studies and we simulated gene expression data as network constrained. Both the two settings consist of 100 regulatory networks. Each regulatory network is composed by one transcription factor (TF) that regulates 10 genes resulting in a total of 1100 genes. Detailed settings are given below.

#### Scenario 1: Not-overlapped networks

The first setting simulates a scenario with not-overlapped networks, which means that the 100 regulatory networks are disjoint each other and each gene is linked to only one TF. Under this assumptions, the degree *d*_*i*_ of each TF = 10 and *d*_*i*_ = 1 for the regulated genes. The edges' weight *w*_*ij*_ = 1 between the TFs and their regulated genes, *w*_*ij*_ = 0 otherwise. The expression value of each TF was generated from a normal standard distribution. The expression values of the ten regulated genes were generated from a conditional normal distribution with positive correlation (ρ = 0.7) between the expression of five genes and the corresponding TF, and negative correlation (ρ = −0.7) for the remaining five genes. This simulates the activation or repression of each gene under the effect of the corresponding TF. The failure times were generated from the Cox model

$$\lambda (t|X)=\lambda_{0}(t)\exp(\sum_{j=1}^{88}\beta_{j}X_{j})$$

which includes only s = 88 relevant genes (i.e., eight regulatory networks). The baseline hazard function λ_0_(*t*) was specified by a Weibull distribution with shape parameter 5 and scale parameter 2. Censoring times were generated from *U*(2, 15) with a censoring rate of about 30%. The sample size was fixed at *n* = 200 and the simulation were replicated 100 times. In this setting of not-overlapped genes, the coefficients β_*j*_, *j*=1, …, 44 were generated from the uniform distribution *U*(0.1, 1), while β_*j*_, *j*=45, …, 88 were generated from *U*(−1.5, −0.1).

For each of the settings above, we quantified the noise as the mean between the variance of each transcription factor (TF) and the variance of the 10 corresponding regulated genes.

#### Scenario 2: Overlapped networks

The second setting simulates a scenario with overlapped networks, where four regulatory networks (i.e., 44 genes) are connected to the other four networks. This mimics the fact that some genes can belong to different pathways regulating different biological processes, as often observed in cancer. For the sake of simplicity, we assume that all the genes (including the TF) in the networks *P*_3_, *P*_4_, *P*_5_, and *P*_6_ are connected to the genes in the remaining four network *P*_1_, *P*_2_, *P*_7_, and *P*_8_ which are maintained disjointed and independent each other. The expression values of the TFs and the regulated genes were generated from a multivariate normal distribution with $cov\left(X_{i},X_{j}\right)=0.5^{\left|i-j\right|}$. The coefficients β_*j*_, *j* = 1, …, 22, corresponding to *P*1 and *P*2, were generated from the uniform distribution *U*(0.1, 0.5), the coefficients corresponding to the 44 common genes β_*j*_, *j* = 23, …, 66 were generated from *U*(−0.1, 0.1) and the coefficients β_*j*_, *j* = 67, …, 88, corresponding to *P*_7_ and *P*_8_, were generated from the uniform distribution *U*(−1, −0.5). Survival times were generated as reported in the first setting with the same censoring rate.

#### Statistical measures

The performance of each method is summarized by four measures: sensitivity, specificity, number of genes selected, and the Matthews correlation coefficient (MCC). The *sensitivity or true positive rate (TPR)* and *specificity or true negative rate (TNR)* are given by

$$TPR=\frac{TP}{TP+FN},\;TNR=\frac{TN}{TN+FP},$$

where TP, TN, FP, and FN denote the numbers of true positives, true negatives, false positives, and false negatives, respectively. A test with high sensitivity (few false negative) has a low type II error rate, while a test with a high specificity (few false positive) has a low type I error rate. The number of genes selected refers to the genes identified as relevant by each method in the training set. The analysis of these genes gives information on prediction accuracy.

The Matthews correlation coefficient (MCC) is defined as

$$MCC=\frac{TP\times TN-FP\times FN}{\sqrt{\left(TP+FP)(TP+FN)(TN+FP)(TN+FN\right)}}.$$

The MCC measure is an global measure of accuracy, and a larger MCC indicates a better performance.

### Real data applications

We applied the three network methods on different real datasets containing large-scale microarray gene expression measurements from ovarian and breast cancer including survival information (see Table 1) in order to facilitate the detection of molecular biomarker and pathway analysis with clinical utility.

**Table 1:** **Microarray Dataset Summary (OS = overall survival)**.

**Datasets**	**Ref**.	**Sample number**	**Platform**	**Genes number**	**Survival data**	**Cancer type**
GSE26712	Bonome et al., 2008	185	Affymetrix U133A	13104	OS	Ovarian
OV-TCGA	The Cancer Genome Atlas Research Network, 2011	578	Affymetrix U133A	13104	OS	Ovarian
GSE20685	Kao et al., 2011	327	Affymetrix U133Plus2	21686	OS	Breast
GSE7390	Desmedt et al., 2007	198	Affymetrix U133A	13718	OS	Breast

#### Ovarian datasets

We downloaded the first ovarian dataset from NCBI Gene Expression Omnibus as raw .CEL files (Bonome: GSE26712). The data contain gene expression profiling for extensive set of 185 primary ovarian tumors untreated late-stage (III–IV) high-grade (2,3) patients hospitalized at the Memorial Sloan-Kettering Cancer Center between 1990 and 2003. The Affymetrix human U133A microarray platform was used. The second ovarian dataset, the ovarian TCGA, was downloaded from The Cancer Genome Atlas data portal (The Cancer Genome Atlas Research Network, 2011). It was obtained at the gene level (level 3) using the Affymetrix human U133A microarray from 578 samples. All patients were diagnosed with high-grade serous carcinoma and were in an advanced stage. We noted that such datasets are very similar in terms of type of patients, platforms, and cancer disease. Therefore, they can be also used for validation.

#### Breast datasets

The breast cancer microarray datasets were downloaded from NCBI GEO database as raw .CEL files (Kao: GSE20685 and Desmedt: GSE7390). Gene expression profiling of the first dataset was conducted on fresh frozen breast cancer tissue collected from 327 patients diagnosed and treated between 1991 and 2004 at the Koo Foundation Sun-Yat-Sen Cancer Center. Hybridization targets were prepared from total RNA according to the Affymetrix U133 plus 2.0 platform. The second breast cancer dataset was chosen on gene expression profiling of frozen samples from 198 N—systemically untreated patients at the Bordet Institute. It was based on the Affymetrix U133 platform.

#### Preprocessing

All the raw files were processed and normalized by RMA package available in Bioconductor (Gentleman et al., 2004). Between arrays normalization was carried out by using the *preprocessCore* package available in Bioconductor (Gentleman et al., 2004). Survival data (OS, i.e., overall survival), censoring indicator and time to death, for each patients in every dataset were also given (Figure 2, step 1).

#### Cancer genes and related functional networks

Following our previous study (Iuliano et al., 2014), in order to better analyze real datasets, we first applied a biologically inspired size reduction of the dataset, then we built an *a-priori* network information for the type of cancer under investigation (see Figure 2, step 1). For a better focus on genes that are more likely to be relevant in cancer, we selected the high-risk cancer genes using the Human Experimental/Functional Mapper (Huttenhower et al., 2009), which is based on a regularized Bayesian integration system. This mapper provides a *p*-value for each gene describing the significance of the relation between the gene and the disease of interest (breast and ovarian cancer, respectively). In our analysis, we selected only the genes with *p* < 0.05. A summary of the final number of the genes selected from each dataset is reported in Table 2. The network matrices used to test the network-based Cox models in our analysis were also derived from the Human Experimental/Functional Mapper which provides maps describing the genes functional activity and interaction networks in over 200 areas of human cellular biology with information from 30,000 genome-scale experiments. This functional network summarizes information from a variety of biologically informative perspectives: prediction of protein function and functional modules, cross-talk among biological processes, and association of novel genes and pathways with known genetic disorders (Huttenhower et al., 2009). The edges of the network are weighted between [0, 1] and express the functional relation between two genes. Note that the functional linkage network includes more information than Human PPI, frequently used as the network prior knowledge. It is clear that taking into account such biological knowledge helps in identifying significant genes that are functionally related in order to obtain important results biologically interpretable.

**Table 2:** **Significant genes number selected using HEFaIMp tool**.

**Datasets**	**Genes number**
GSE26712	1068
OV-TCGA	1068
GSE20685	536
GSE7390	536

In order to adapt the gene network to the different methods, the final weight matrix was slightly different from method to method. In particular, since *AdaLnet* requires a weight matrix consisting of 0 and 1, each matrix element was set equal to 0 (or 1) if the weight value was below (or above) a fixed threshold equals to 0.5. On the other hand, *Net-Cox* uses the original weight matrix as obtained in the original paper (Huttenhower et al., 2009).

## Results

In our study, we analyzed three network-based Cox regression methods described in Section Methods both on simulated and real data. Here, the major interest is the association of genomic features with clinical outcomes under specific scenarios. Simulation studies were based on two different biological scenarios and were introduced to show the performance of the selected network methods. While, real data analysis was performed in order to provide a better understanding of the outcomes in terms of predictive/prognostic biomarkers and to demonstrate their validity and clinical utility. In particular, we first investigated the three methods in terms of survival prediction performances and then, a pathway analysis was carried out focusing on the relevance in cancer of the selected genes.

It is important to note that the goal of this study is not to provide a rank list of the analyzed methods, but to present a accurate study for the identification of new cancer related genes and core pathways in order to make available such information to biomedical community in the form of a comprehensive methodological procedure (see Figure 1).

### Simulation studies

We analyze the performance of the three analyzed methods in two simulation settings where the number of relevant genes is fixed *a-priori* to 88 genes. The first setting simulates a scenario with not overlapped pathways, which means that each gene in the network belongs to only one pathway (not-overlapped pathways). The second setting represents a more realistic scenario with a set of genes shared among different pathways (overlapped pathways). In both cases, a five-fold cross validation was conducted on the full dataset in order to select the tuning parameters (λ, α) and to obtain the coefficient estimates by using the three methods. The details of the simulation data are reported in Section Methods.

The performance of each method is summarized by several statistical measures: sensitivity, specificity, number of selected genes, false positive rates, and Matthews correlation coefficient (MCC). Simulation results for both the models are reported in Tables 3, 4, respectively (standard deviation is reported in brackets). To analyze the signature genes identified by *Net-Cox*, which is a method based on ridge regression, we considered three different consensus rankings where the number of significant genes selected by the method was fixed to 44, 88, and 176 genes, respectively. The selected genes were classified in descending order according to the absolute value of the regression coefficients. On the other hand, to better highlight the variable selection performance of *AdaLnet* and *fastcox*, we split the 100 iterations in two groups based on the number of genes selected at each iteration. We fixed 100 genes as threshold for *AdaLnet* and 10 genes for *fastcox*, then we computed again the statistical measures based on the two groups.

**Table 3:** **Simulation results for Not-Overlapped settings. Sensitivity, specificity, number of selected genes, false positive rates, and MCC were averaged over the 100 replications**.

	**Sensitivity**	**Specificity**	**No. genes**	**No. FP**	**MCC**
**Net-Cox**					
No. genes = 44	0.240 (0.042)	0.977 (0.004)	44.000 (0.000)	22.910 (3.677)	0.300 (0.063)
No. genes = 88	0.489 (0.071)	0.956 (0.006)	88.000 (0.000)	44.940 (6.233)	0.445 (0.077)
No. genes = 176	0.737 (0.087)	0.890 (0.008)	176.000 (0.000)	111.180 (7.692)	0.464 (0.070)
**AdaLnet**					
General setting	0.444 (0.250)	0.792 (0.170)	249.360 (193.786)	210.330 (172.384)	0.190 (0.059)
No. genes ≤ 100	0.200 (0.085)	0.967 (0.021)	51 (27.256)	33.395 (21.227)	0.220 (0.064)
No. genes > 100	0.627 (0.160)	0.660 (0.099)	399 (113.254)	343.807 (100.118)	0.166 (0.041)
**fastcox**					
General setting	0.141 (0.117)	0.970 (0.037)	42.62 (46.613)	30.19 (37.833)	0.160 (0.082)
No. genes ≤ 10	0.017 (0.017)	0.999 (0.0002)	1.524 (1.486)	0.048 (0.216)	0.099 (0.07)
No. genes > 10	0.231 (0.063)	0.949 (0.036)	72.379 (40.331)	52.017 (36.492)	0.204 (0.054)

*The table reports three consensus rankings for Net-Cox obtained selecting 44, 88, and 176 genes. For AdaLnet and fastcox, we show the results related to the general setting, and the statistical measures obtained when the number of selected genes is higher (or lower) of a fixed threshold (threshold was set equal to 100 for AdaLnet and equal to 10 for fastcox). Standard deviation is reported in brackets*.

**Table 4:** **Simulation results for overlapped settings**.

	**Sensitivity**	**Specificity**	**No. genes**	**No. FP**	**MCC**
**Net-Cox**					
No. genes = 44	0.156 (0.043)	0.970 (0.004)	44.000 (0.000)	30.240 (3.766)	0.175 (0.064)
No. genes = 88	0.288 (0.044)	0.938 (0.004)	88.000 (0.000)	62.620 (3.842)	0.227 (0.048)
No. genes = 176	0.386 (0.044)	0.860 (0.003)	176.000 (0.000)	142.010 (3.860)	0.182 (0.035)
**AdaLnet**					
General Setting	0.262 (0.178)	0.879 (0.144)	145.280 (160.666)	122.240 (145.679)	0.166 (0.067)
No. genes ≤ 100	0.141 (0.064)	0.977 (0.020)	35.635 (24.760)	23.206 (20.296)	0.196 (0.060)
No. genes > 100	0.467 (0.106)	0.713 (0.105)	331.973 (114.325)	290.865 (106.135)	0.114 (0.043)
**fastcox**					
General setting	0.098 (0.099)	0.974 (0.039)	34.55 (47.732)	25.89 (39.807)	0.134 (0.061)
No. genes ≤ 10	0.019 (0.015)	0.999 (0.0001)	1.679 (1.281)	0.0178 (0.134)	0.115 (0.065)
No. genes > 10	0.199 (0.061)	0.942 (0.040)	76.386 (45.224)	58.818 (40.830)	0.158 (0.044)

*Sensitivity, specificity, number of selected genes, false positive rates and MCC were averaged over the 100 replications. The table reports three consensus rankings for Net-Cox obtained selecting 44, 88, and 176 genes. For AdaLnet and fastcox, we show the results related to the general setting, and the statistical measures obtained when the number of selected genes is higher (or lower) that a fixed threshold (threshold was set equal to 100 for AdaLnet and equal to 10 for fastcox). Standard deviation is reported in brackets.Standard deviation is reported in brackets*.

In the not-overlapped setting, *Net-Cox* performed better than the other two methods as showed by the MCC, which provides an overall measure of accuracy. In particular, when considering 44 and 88 genes, the false positive rate in *Net-Cox* was 22.910 and 44.940, respectively, with MCC equals to 0.300 and 0.445. Sensitivity and specificity were, respectively, 0.240 and 0.977 in the first case, 0.489 and 0.956 in the second case study. When the number of selected genes was increased to 176, even if the false positive rate increased resulting in a lower specificity (0.890), the sensitivity reached its highest values producing the highest MCC (0.464).

Since the majority of the selected genes were irrelevant and both *AdaLnet* and *fastcox* resulted in sparse models, specificity was much higher than sensitivity and was comparable between the two variable selection methods. In particular, in the not-overlapped setting, *AdaLnet* selected in average 249.360 genes with a false positive rate equals to 210.330. Sensitivity and specificity were equal to 0.444 and 0.792 resulting in a MCC of 0.190. On the other hand, *fastcox* selected in average 42.62 genes with a false positive rate of 30.19. MCC was equal to 0.160 with sensitivity 0.141 and specificity 0.970.

*AdaLnet* had the best performance when the number of selected genes was below 100, while *fastcox* exhibit the best performance when the number of genes was above 10. This means that in the other cases the methods fail in the execution of the cross-validation (see Supplementary Image 1).

In the overlapped-pathways setting, *Net-Cox* obtained the highest MCC overall when considering 88 genes (MCC equals to 0.227) with a false positive rate equals to 62.620, sensitivity 0.288 and specificity 0.938. However, even if the specificity levels of the three consensus rankings were almost equal to the previous setting (specificity for 44, 88, and 176 genes equals to 0.970, 0938, and 0.860, respectively), in this setting *Net-Cox* sensitivity decreased resulting in lower MCC compared to the not-overlapped case (MCC for 44, 88, and 176 genes equals to 0.175, 0.227, and 0.182, respectively). *AdaLnet* and *fastcox* also reported lower MCCs compared to the not-overlapped setting (MCC equals to 0.166 in *AdaLnet* and 0.134 in *fastcox*). In particular, both *AdaLnet* and *fastcox* showed an higher specificity than before (0.879 and 0.974, respectively) but a lower sensitivity (0.262 and 0.098). Further analysis showed that *AdaLnet* had the highest MCC when the number of selected genes was below 100 (MCC 0.196), while *fastcox* had the highest MCC (0.158) when the number of selected genes was above 10, in accordance with the previous results (see Supplementary Image 2).

### Real data analysis

In order to evaluate the performance of the three Cox models in terms of survival analysis, we used cross-validated Kaplan–Meier curves (Simon et al., 2011b) for overall survival (OS) both on ovarian and breast microarray studies (see Figure 2, step 2). Note that *p*-value was estimated within the same dataset but the cross-validation approach is used to correct over optimistic conclusions due to the lack of independence between samples.

Moreover, since the ovarian datasets are comparable in terms of types of patients, platforms and cancer disease, Kaplan–Meier curves and two-side log-rank test were used to estimate the survival time and stratify the low-risk and high-risk groups on the independent test set (see Figure 2, step 3).

Table 5 reports the number of genes selected by the three Cox regression methods for each OS and the optimal tuning parameter α. Interestingly, the optimal α was often equal to 0.5, indicating that there was a good balance between statistical constraints and network information. These results confirm that the network carries important information useful for improving survival analysis. Moreover, since *Net-Cox* is a method based on ridge regression, the genes are only shrunk and it is necessary to fix a threshold for selecting the most relevant cancer genes. Hence, within each fold, we ordered the genes according to the absolute value of the corresponding regression coefficients, then we considered the union of the top 50 genes selected in each fold.

**Table 5:** **Optimal α cross-validated value calculated on the ***k*** training sets**.

**Datasets**	***k* Partitions**	**Net-Cox**		**AdaLnet**		**fastcox**	
		**α**	**Genes selected**	**α**	**Genes selected**	**α**	**Genes selected**
GSE26712	5	0.2	101	0.5	23	0.01	453
OV-TCGA	5	0.5	99	0.5	38	0.1	623
GSE20685	5	0.5	76	0.5	28	0.01	298
GSE7390	5	0.5	89	0.5	14	0.01	423

In the following, we present the main results obtained.

#### Results on the ovarian datasets

**Figure 3:**
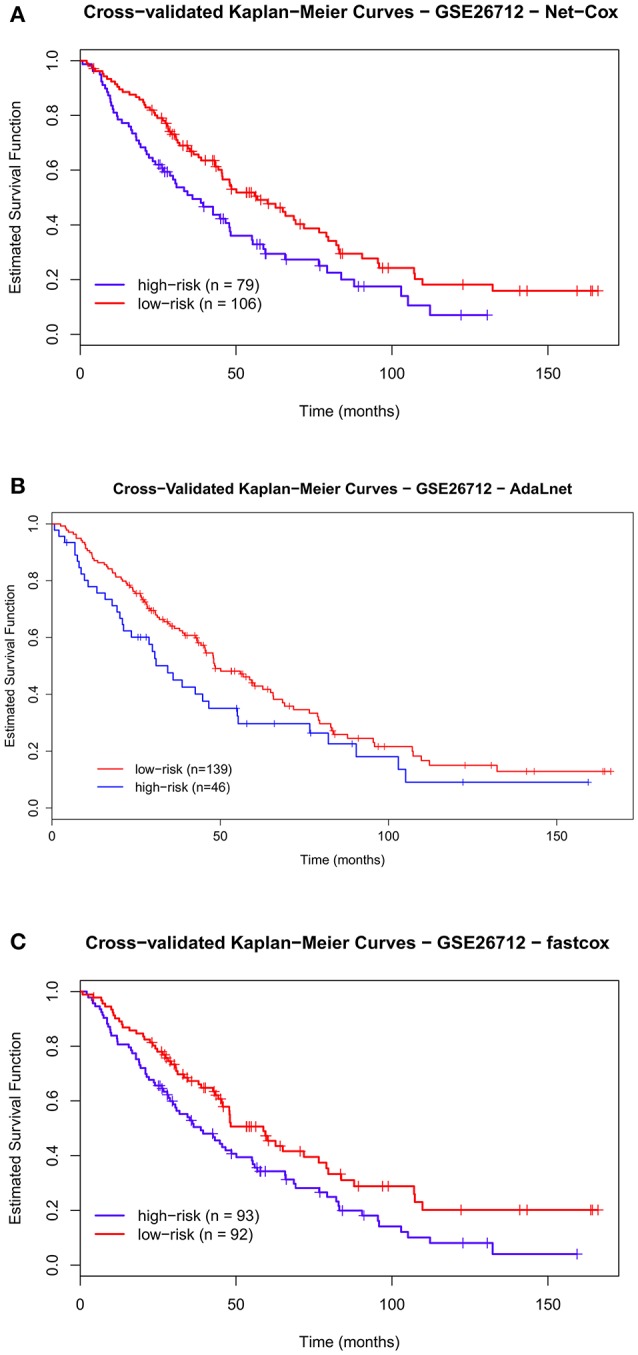
**Cross-validated Kaplan–Meier curves of the prognostic models on GSE26712 dataset**. The patients are divided in *high-risk* and *low-risk groups* based on the pathways and genes selected by each methods for overall survival (OS). The survival probabilities of these two groups are compared using the log-rank test by using *Net-Cox* **(A)**, *AdaLnet* **(B)**, and *fastcox* **(C)**.

**Figure 4:**
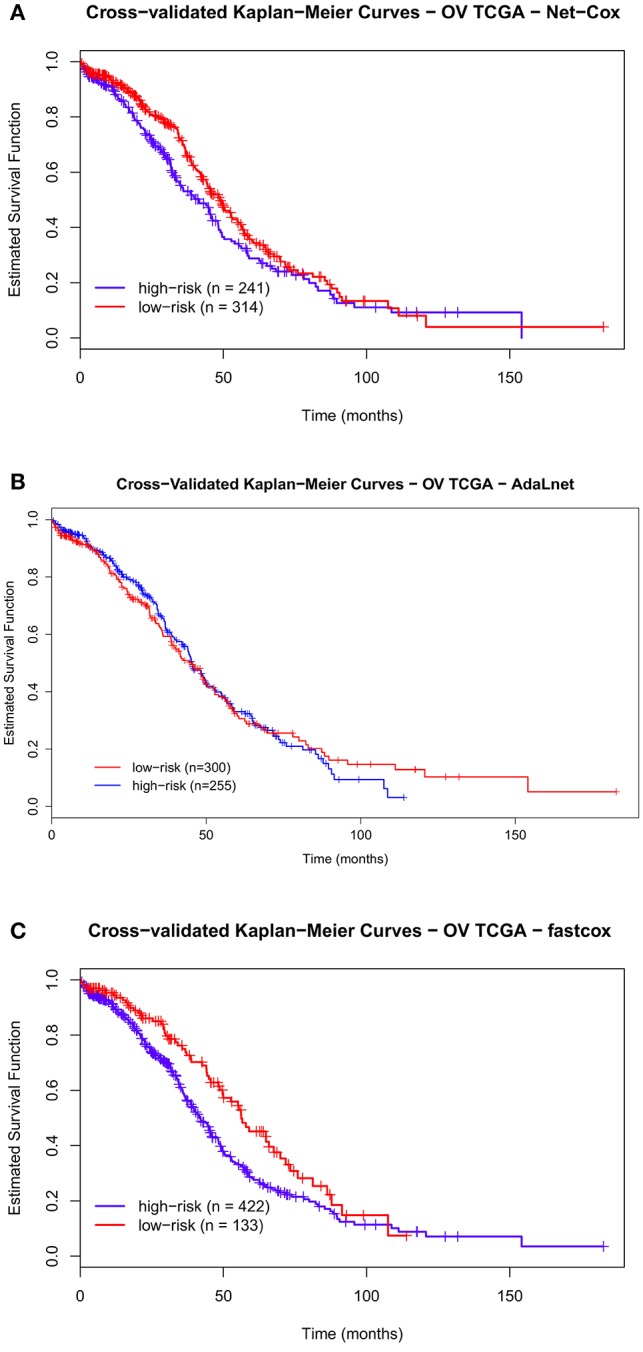
**Cross-validated Kaplan–Meier curves of the prognostic models on OV TCGA dataset**. The patients are divided in *high-risk* and *low-risk groups* based on the pathways and genes selected by each methods for overall survival (OS). The survival probabilities of these two groups are compared using the log-rank test by using *Net-Cox* **(A)**, *AdaLnet* **(B)**, and *fastcox* **(C)**.

Figures 3, 4 show the cross-validated Kaplan–Meier curves for *high-and-low risk groups* patients selected in the ovarian datasets (Benome: GSE26712 and OV TCGA datasets, respectively). Figure 3 shows that in the Bonome dataset the gap between the survival curves of the two risk groups in *Net-Cox* (Figure 3A) and *fastcox* (Figure 3C) is wider compared to *AdaLnet* (Figure 3B). In particular, in predicting survival probabilities, *fastcox* (permuted *p* < 0.05) seem to discriminate the risk groups better than *Net-Cox* and *AdaLnet* where the permuted *p* > 0.05. These findings confirm the results previously obtained in Iuliano et al. (2014), in relation to the survival curves for each method. This was mainly due to the cross-validation approach used in this analysis to overcome the sample splitting problem with too small dataset.

On the other hand, in the OV TCGA dataset (Figure 4), the survival curves for *high-and-low risk* patients are not significantly separated. In particular, *fastcox* is the only method with a significant difference (permuted *p* < 0.05) in the OS between the *high-and-low-risk groups*.

**Figure 5:**
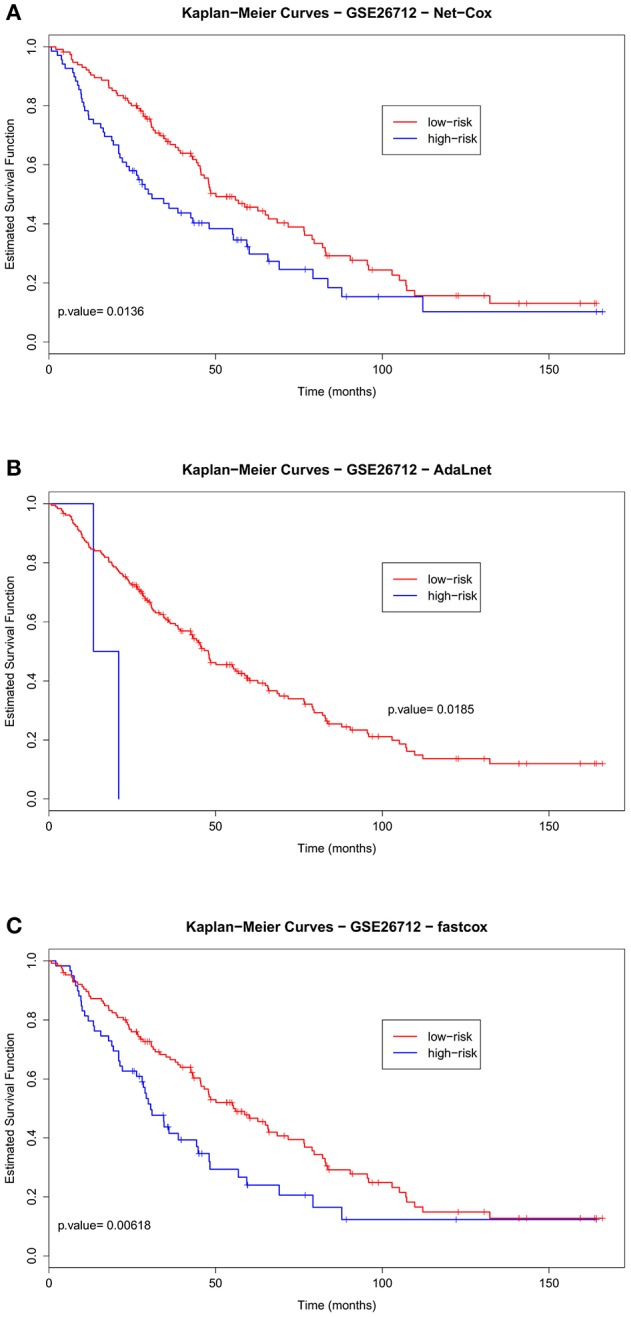
**Kaplan–Meier curves for validation test on GSE26712 ovarian dataset**. The curves show the patients stratified by using the genes selected in the OV TCGA dataset by *Net-Cox, AdaLnet*, and *fastcox* [**(A)**, **(B)**, and **(C)**, respectively] with threshold *p* < 0.05.

Finally, to test the survival prediction across independent datasets, we used the ovarian OV TCGA dataset as training set, and the Benome dataset as the test set to predict the risk scores of the patients (see Figure 2, step 3). Figure 5 shows the Kaplan–Meier curves for the two risk groups (high-and-low risk groups) in the Benome dataset obtained by *Net-Cox* (Figure 5A), *AdaLnet* (Figure 5B), and *fastcox* (Figure 5C). All the three methods gave a significant *p*-value at the 5% significance level (log-rank test, *p* < 0.05).

#### Results on breast datasets

**Figure 6:**
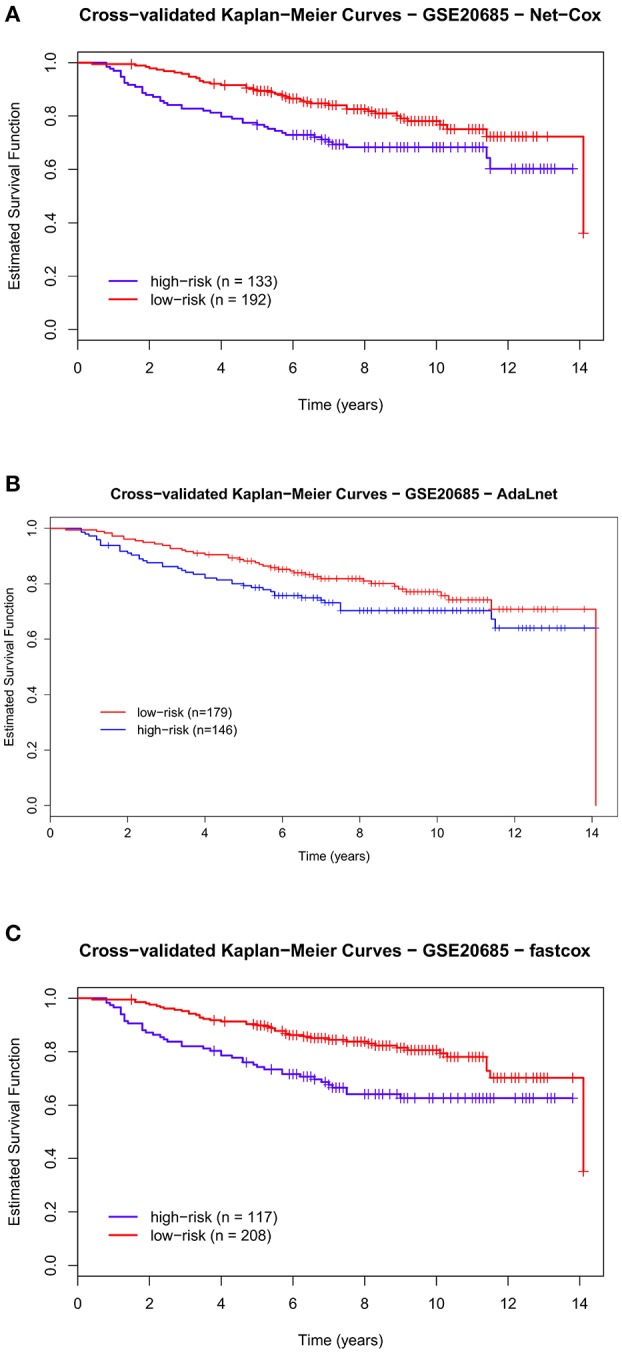
**Cross-validated Kaplan–Meier curves of the prognostic models on GSE20685 dataset**. The patients are divided in *high-risk* and *low-risk groups* based on the pathways and genes selected by each methods for overall survival (OS). The survival probabilities of these two groups are compared using the log-rank test by using *Net-Cox* **(A)**, *AdaLnet* **(B)**, and *fastcox* **(C)**.

**Figure 7:**
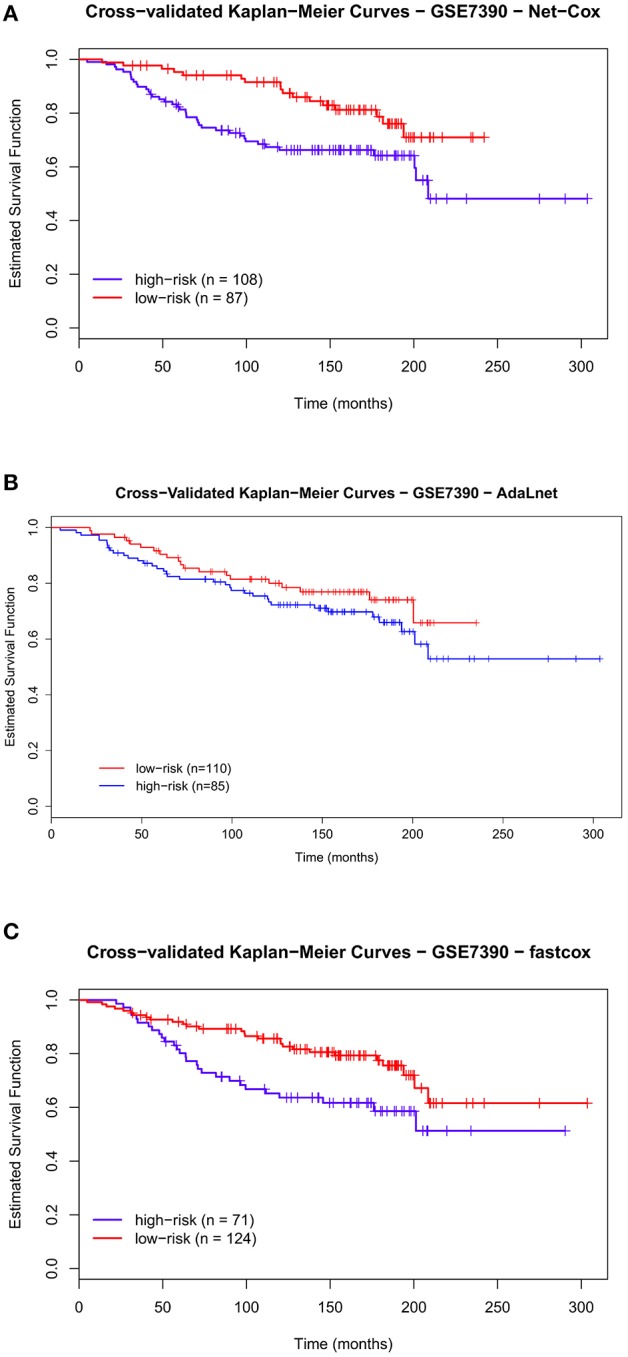
**Cross-validated Kaplan–Meier curves of the prognostic models on GSE7390 dataset**. The patients are divided in *high-risk* and *low-risk groups* based on the pathways and genes selected by each methods for overall survival (OS). The survival probabilities of these two groups are compared using the log-rank test by using *Net-Cox* **(A)**, *AdaLnet* **(B)**, and *fastcox* **(C)**.

Figures 6, 7 show the cross-validated Kaplan–Meier curves for *high-and-low risk groups* patients selected in the breast datasets (Kao: GSE20685 and Desmedt: GSE7390, respectively). In the Kao dataset, the permuted *p*-value related to Figure 6A (*Net-Cox*) and Figure 6C (*fastcox*) was smaller than 0.05, which means the *high-risk* and *low-risk* groups were significantly separated and the selected pathways and genes were related to survival times. In Figure 6B (*AdaLnet*), a patient of the high-risk group fell in the low-risk group and the permuted *p*-value is not significant.

We performed the same analysis for *high-and-low risk* patients in the Desmedt dataset. Also in this case, there was a significant difference in OS between the two risk groups as shown in Figure 7A (*Net-Cox*) and Figure 7C (*fastcox*) where the permuted *p*-value is smaller than 0.05. In Figure 7B (*AdaLnet*) the permuted *p*-value is not significant.

### Identified pathways

In this section, we present the results of the analysis in terms of KEGG pathways analysis based only on *not-isolated* genes (see section Methods for details). We report here only the networks related to *AdaLnet* and *Net-Cox* since all the networks related to *fastcox* have more than 100 node and 2000 edges and a clear visualization would not be possible. However, the lists of the genes selected by *fastcox* and the related pathways are reported in Supplementary Table 1 (ovarian datasets) and Supplementary Table 2 (breast datasets).

**Figure 8:**
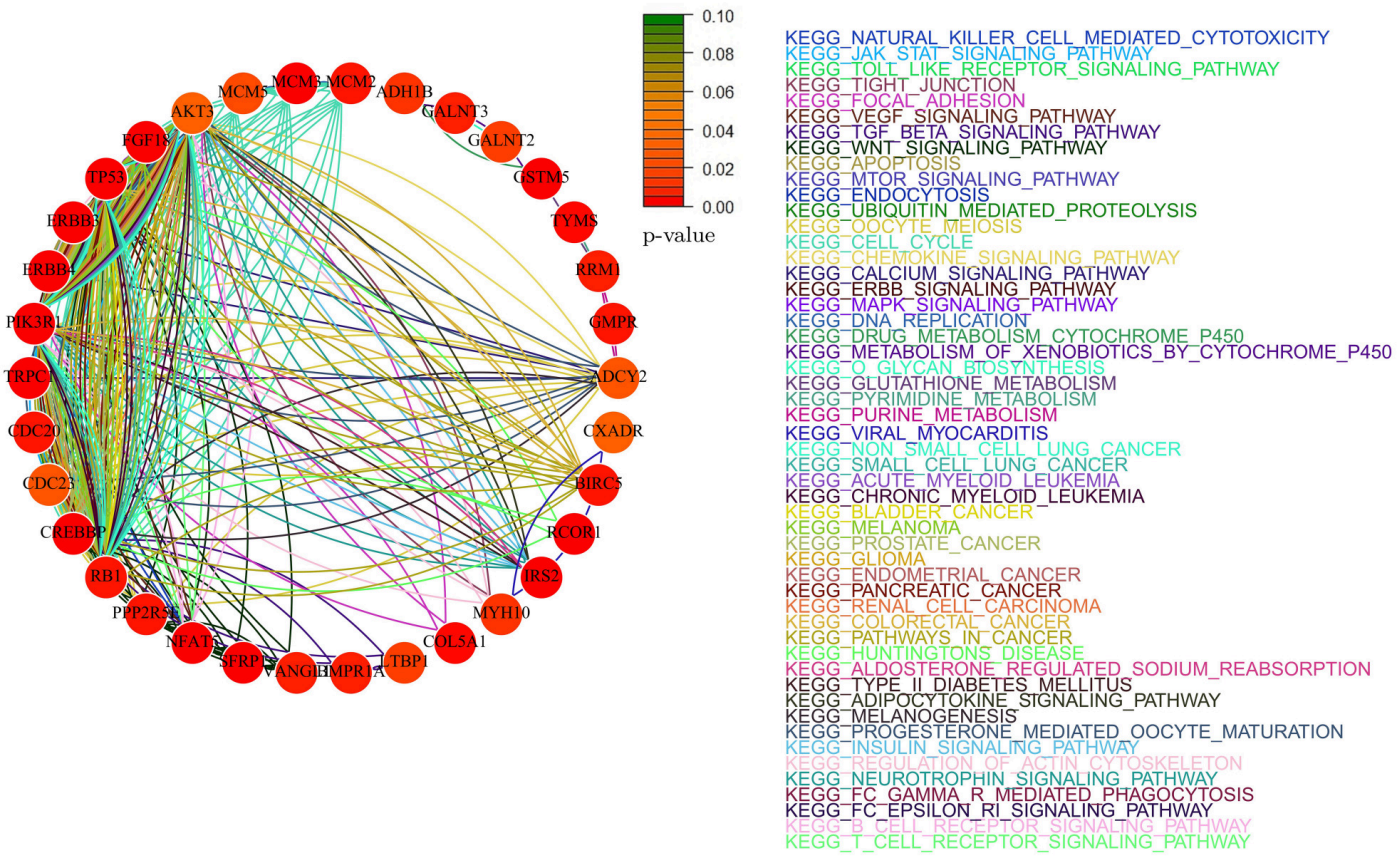
**Gene-network of not isolated genes selected by ***Net-Cox*** in the Bonome ovarian dataset (GSE26712)**. Each node represents a gene and an edge between two nodes means that the two genes belongs to the same pathway. Different colors are used for different pathways. The color of each node represents the *p*-value of the interaction between the gene and ovarian cancer (Huttenhower et al., 2009). Genes with *p* > 0.10 are represented in green.

**Figure 9:**
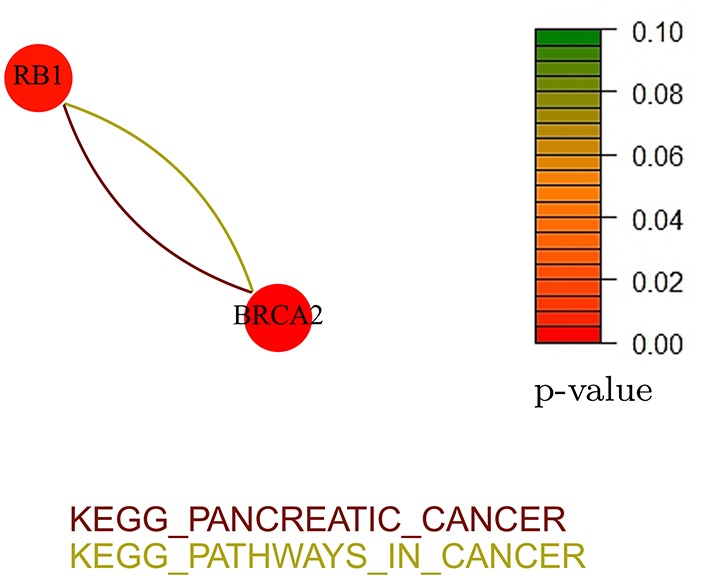
**Gene-network of not isolated genes selected by ***Adalnet*** in the Bonome ovarian dataset (GSE26712)**. Each node represents a gene and an edge between two nodes means that the two genes belongs to the same pathway. Different colors are used for different pathways. The color of each node represents the *p*-value of the interaction between the gene and ovarian cancer (Huttenhower et al., 2009). Genes with *p* > 0.10 are represented in green.

Figures 8, 9 show the gene-networks obtained for the Bonome dataset (GSE26712) built on the genes identified by *Net-Cox* and *Adalnet*, respectively. From the color of the nodes, we can infer that all the selected genes have a significant relation with ovarian cancer. Indeed, almost all the genes are close to red except for *AKT3* which has a *p*-value correlation equal to 0.039. Indeed, *AKT3* is usually involved in prostate and breast cancer (Nakatani et al., 1999). However, since it was selected both by *Net-Cox* and *fastcox*, a possible significant relation between *AKT3* and ovarian cancer could be inferred as indeed confirmed by literature (Liby et al., 2012). In particular, *AKT3* has a specific role in the genesis of ovarian cancer through modulation of G2-M phase transition (Cristiano et al., 2006). As showed in Figure 8, *AKT3* is also involved in many cancer pathways, such as *KEGG basal cell carcinoma, KEGG prostate cancer*, and *KEGG melangiogenesis*. It is worthy to note that this gene was also selected in our previous study (Iuliano et al., 2014) by all the analyzed methods and it was also involved in the same cancer related pathways. These findings confirm the importance of *AKT3* in ovarian cancer as confirmed indeed by literature (Cristiano et al., 2006).

In the Bonome dataset (GSE26712), *Adalnet* selected only two *not-isolated* genes (*RB1* and *BRCA2*) involved in two different cancer pathways (Figure 9). Both the genes have been frequently observed in epithelial ovarian cancer (Flesken-Nikitin et al., 2003; Dinulescu et al., 2005; Naora and Montell, 2005) and several studies report their stable correlation (Flesken-Nikitin et al., 2003; The Cancer Genome Atlas Research Network, 2011). Moreover, the strong interaction between *RB1* and the tumor protein *TP53* (Dong et al., 1997; Schuijer and Berns, 2003) has been identified by *Net-Cox* and *fastcox* (Figure 8).

**Figure 10:**
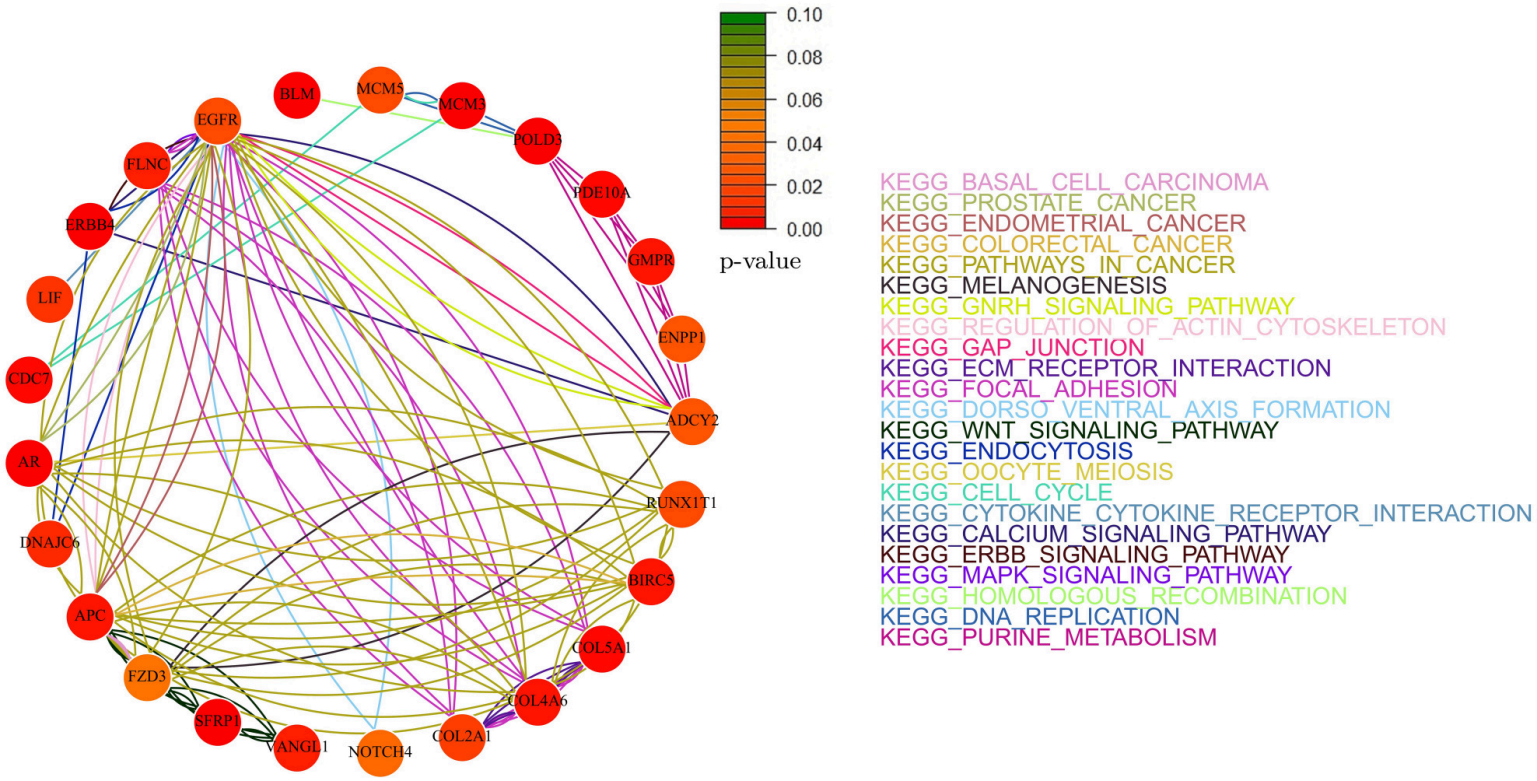
**Gene-network of not isolated genes selected by ***Net-Cox*** in the TCGA ovarian dataset**. Each node represents a gene and an edge between two nodes means that the two genes belongs to the same pathway. Different colors are used for different pathways. The color of each node represents the *p*-value of the interaction between the gene and ovarian cancer (Huttenhower et al., 2009). Genes with *p* > 0.10 are represented in green.

**Figure 11:**
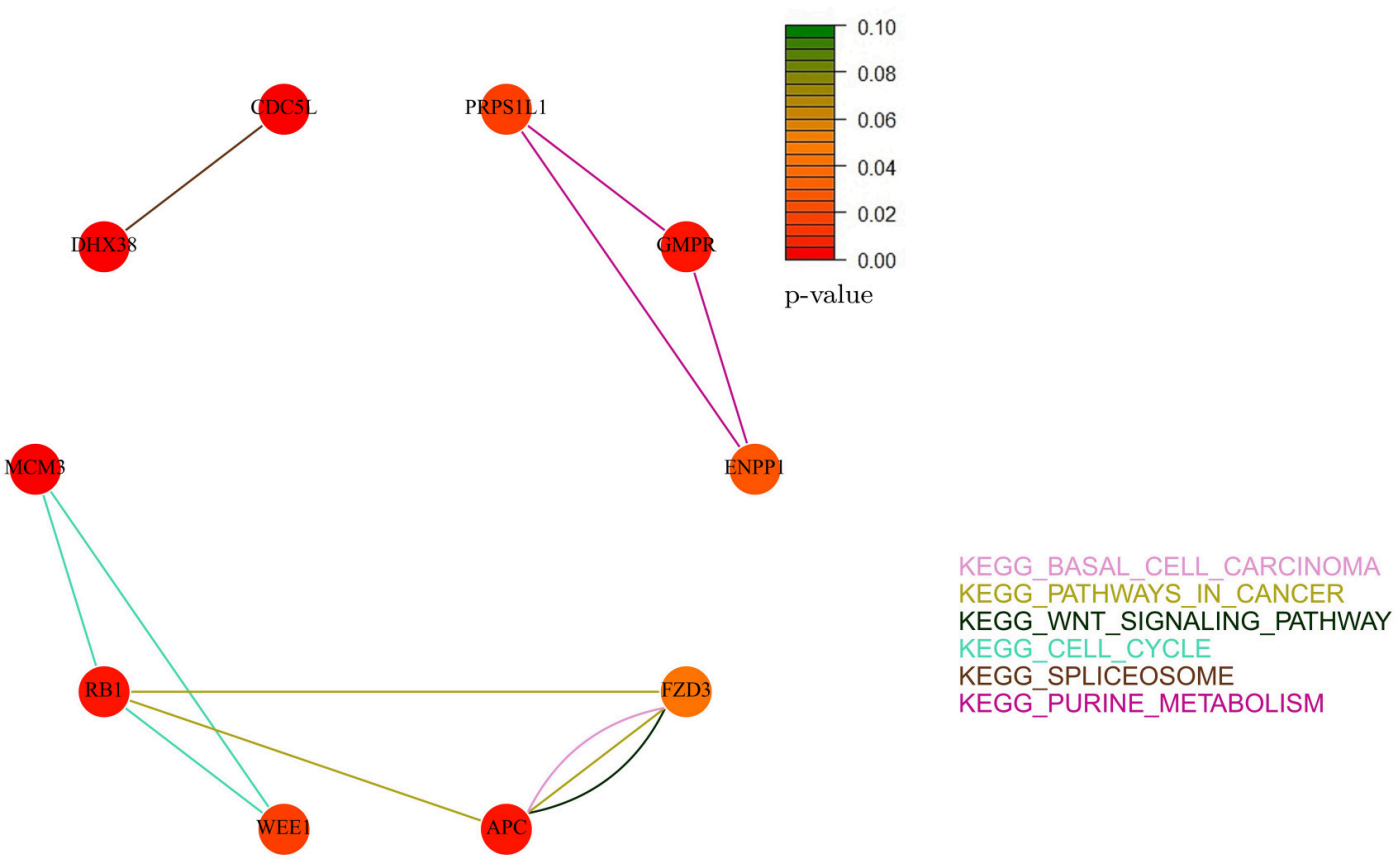
**Gene-network of not isolated genes selected by ***Adalnet*** in the TCGA ovarian dataset**. Each node represents a gene and an edge between two nodes means that the two genes belongs to the same pathway. Different colors are used for different pathways. The color of each node represents the *p*-value of the interaction between the gene and ovarian cancer (Huttenhower et al., 2009). Genes with *p* > 0.10 are represented in green.

Figures 10, 11 show the gene-networks obtained for the OV TCGA ovarian dataset built on the genes identified by *Net-Cox* and *Adalnet*, respectively. As already observed in the Bonome dataset analysis, all the selected genes in the OV TCGA dataset resulted strongly correlated with ovarian cancer. Indeed, almost all the genes are close to red. The only gene with a slightly different color is *FZD3* which has a *p*-value of 0.049 and was selected by all the three methods. Hence, even if this gene has been mainly classified as gastric-cancer-related (Katoh, 2005), our results prove that it also has a relevant effect in ovarian cancer as confirmed by literature (Tapper et al., 2001). It is also important to note that other genes have been selected by all the three methods (i.e., *GMPR, ENPP1*, and *APC*). Such genes have been already classified as ovarian-related in cancer literature (Gayther et al., 1997; Kikuchi et al., 2007; Rikova et al., 2007), but, in our analysis, the pathways involved in such relation are also investigated. For example, while *GMPR* and *ENPP1* interact simply through the *KEGG purine metabolism* pathway, the *APC*-*FZD3* interaction involves three different pathways: *KEGG basal carcinoma, KEGG pathways in cancer*, and *KEGG wnt signaling pathway*.

It is worthy to note that some of the genes selected by the three methods (e.g., *NPY, COL5A1, EGFR*, and *FBL1*) have been already reported in literature (Zhang et al., 2013) where an analysis of subnetwork signatures in ovarian cancer based on Cox model is presented. Moreover, our approach selected new genes, such as *AKT3* and *RB1*, which are also related to ovarian cancer (Flesken-Nikitin et al., 2003; Cristiano et al., 2006). These results show that our findings are consistent with the previous ones including, at the same time, other gene signatures.

**Figure 12:**
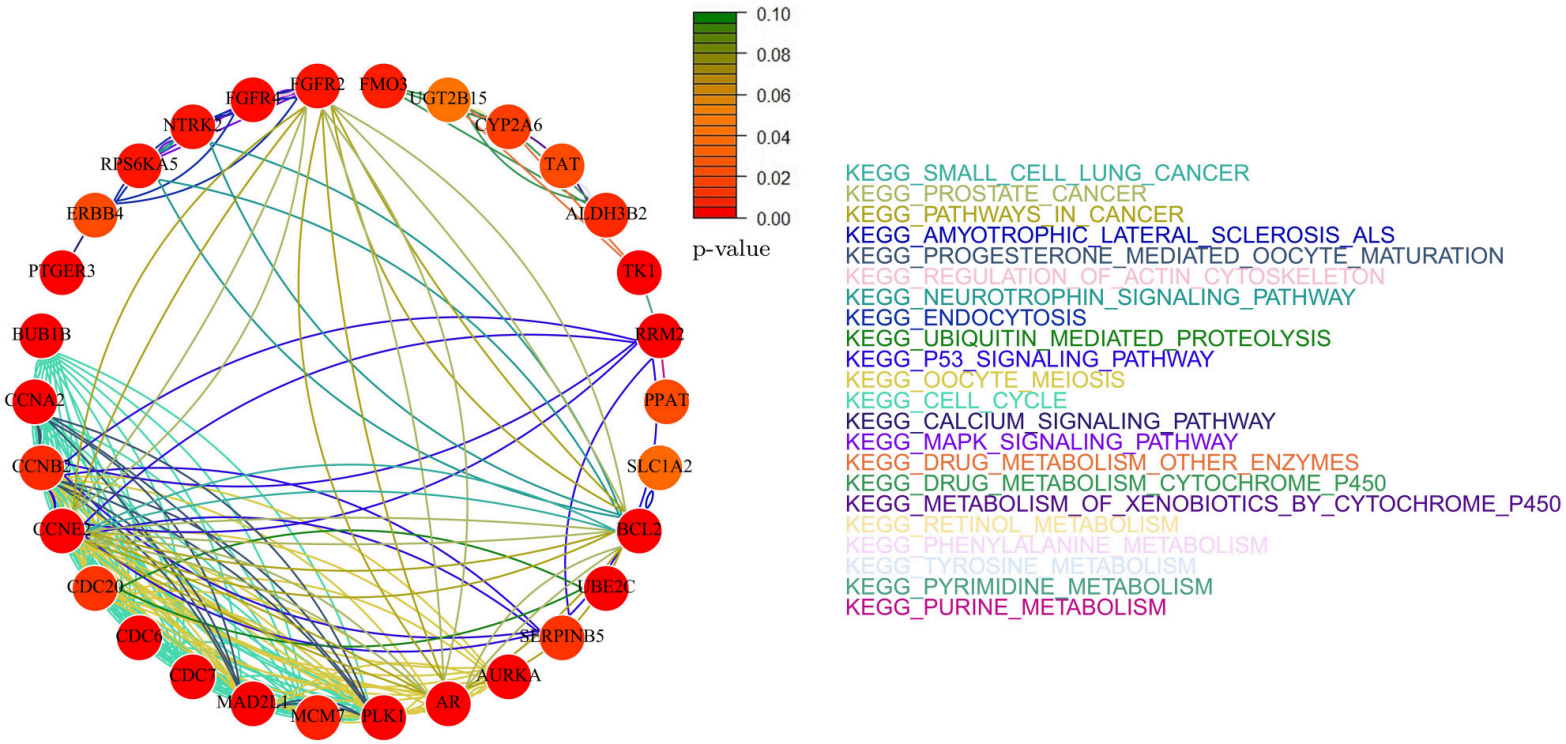
**Gene-network of not isolated genes selected by ***Net-Cox*** in the GSE20685 breast dataset**. Each node represents a gene and an edge between two nodes means that the two genes belongs to the same pathway. Different colors are used for different pathways. The color of each node represents the *p*-value of the interaction between the gene and breast cancer (Huttenhower et al., 2009). Genes with *p* > 0.10 are represented in green.

**Figure 13:**
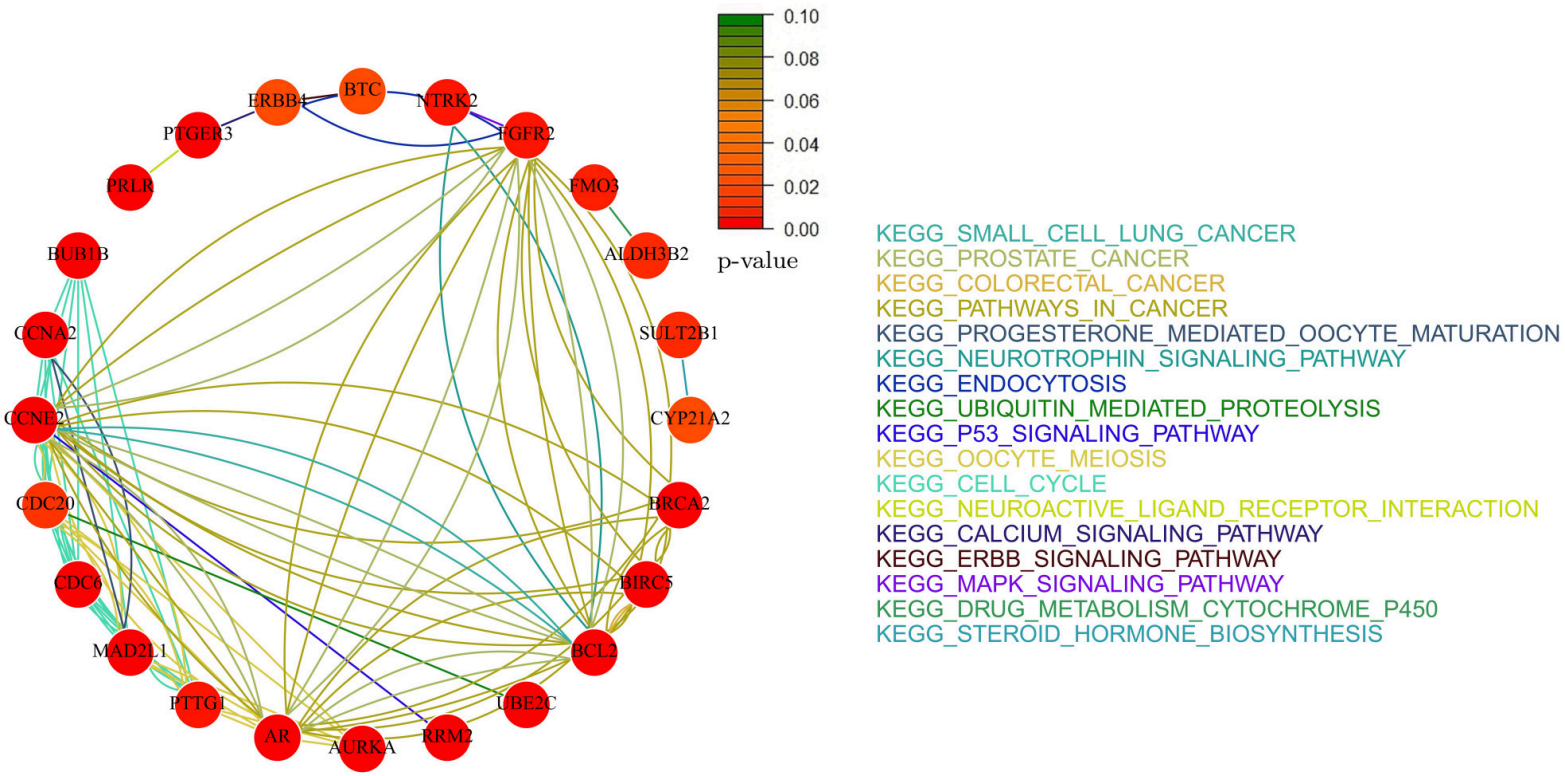
**Gene-network of not isolated genes selected by ***Adalnet*** in the GSE20685 breast dataset**. Each node represents a gene and an edge between two nodes means that the two genes belongs to the same pathway. Different colors are used for different pathways. The color of each node represents the *p*-value of the interaction between the gene and breast cancer (Huttenhower et al., 2009). Genes with *p* > 0.10 are represented in green.

Figures 12, 13 report the gene-networks selected in the Kao dataset (GSE20685) by *Net-Cox* and *Adalnet*, respectively. *FGFR2* and *BCL2* were again selected in this dataset confirming the strong relevance of the two genes in breast cancer. Moreover, *BRCA2* (Wooster et al., 1995) was selected by *Net-Cox* and *fastcox* confirming the accuracy of our analysis. It is also worthy to note that in all the breast cancer gene-networks the *KEGG prostate cancer* is always recurrent. This is mainly due to the common biomarkers between the two diseases (Yang et al., 1998; Mattie et al., 2006) and through our analysis new common biomarkers can be identified.

**Figure 14:**
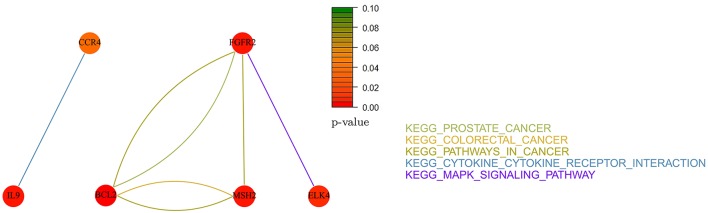
**Gene-network of not isolated genes selected by ***Net-Cox*** in the GSE7390 breast dataset**. Each node represents a gene and an edge between two nodes means that the two genes belongs to the same pathway. Different colors are used for different pathways. The color of each node represents the *p*-value of the interaction between the gene and breast cancer (Huttenhower et al., 2009). Genes with *p* > 0.10 are represented in green.

In the Desmedt dataset (GSE7390), all the genes selected by *Adalnet* were *isolated* and no network was built in this case. A list of the genes selected is reported in Table 6. Figure 14 reports the gene-network related to the genes selected by *Net-Cox*. All the selected genes show a strong relation with the disease, such as *FGFR2* and *BCL2*, which were selected by both *Net-Cox* and *fastcox* and are involved in *KEGG prostate cancer* and in *KEGG pathways in cancer*. Both the genes are largely known as independent prognostic marker in breast cancer (Hunter et al., 2007; Thomadaki et al., 2007; Callagy et al., 2008). Both *Net-Cox* and *fastcox* selected *UGT2B15*, which has a breast-cancer-correlation *p* = 0.049. This gene has been usually involved in prostate cancer (Gsur et al., 2002), but recent works highlight its role also in breast cancer (Wegman et al., 2007).

**Table 6:** **List of genes selected by ***Adalnet*** in the breast dataset GSE7390**.

**Genes**	***p*-values**
BRCA1	0
GYPB	0.0489
MYBL2	0.0026
ADH6	0.0259
GHRHR	0.0007
GUCY2C	0.0323
PPP2R1B	0.0321
SLC1A2	0.0450
SLC12A3	0.0483
LIPF	0.0449
TRIP13	0.0001
PPM1E	0.0026
CEP152	0.0064
PSPC1	0.0475

*The second column reports the breast-cancer correlation p-value of each gene accordingly (Huttenhower et al., 2009). All the selected genes resulted isolated and no network was built in this case*.

In the analysis of the breast datasets, there was no overlap with our previous study (Iuliano et al., 2014). This was mainly due to the different datasets analyzed here potentially (different cancer subtype and different types of conditions) and to the more sophisticated procedures followed in this analysis. Indeed, in our previous work, we split the dataset in training and test set only once, while here we used a cross-validation procedure that is expected more robust results.

## Discussion and conclusions

A key issue in cancer survival analysis is uncovering the relation between gene expression profiles and cancer patients survival in order to identify biomarkers for disease diagnosis and treatment. In the last years, there has been a growing interest in methods that incorporate network information into classification algorithms for genes signature discovery. The main aims are to identify molecular biomarkers that reliably predict patient's response to therapy and to avoid ineffective treatment for reducing drug side-effects and associated costs. For this purpose, prognostic and diagnostic biomarker signatures need to be derived from omics data for various disease entities in order to offer useful methodological and practical strategy in research and clinical settings.

Here, we presented an extended methodological strategy for the analysis of gene signatures and survival prediction (see Figure 1). We integrated a new cross-validation method (Simon et al., 2011b) with the most recent network penalized Cox models (Yang and Zou, 2012; Zhang et al., 2013; Sun et al., 2014) to obtain an effective multi-splitting of the data and achieve an accurate survival prediction (see Figure 2). The analysis of the models was based both on simulated and real datasets in order to provide an accurate analysis in terms of statistical and biological investigation. Indeed, we showed that, given a number of variables not extremely high, all the analyzed methods were able to select the altered genes under different simulation settings. On the other hand, the analysis on real cancer datasets showed that through the integration of network information into Cox regression methods it is possible to identify cancer gene signatures with an accurate prognostic performance. Therefore, the contribution of this study is two-fold. Firstly, to obtain an integrative analysis of cancer genes networks and survival prediction. Secondly, to provide a computational and methodological framework for better investigating cancers regulatory networks and facilitating the management of patients in terms of prognosis, diagnosis and treatment.

The findings of this study have a number of important implications for future practice. Firstly, a practically appealing study based on a fast screening procedure (Fan and Lv, 2008; Fan et al., 2010) could be introduced in order to reduce the size of the feature space to a moderate scale. In fact, several types of screening procedures could be combined to integrate biological information into statistical screening analysis and provide more definitive understanding of the gene-regulatory networks. Secondly, the integration of clinical information and data from different omics (e.g., epigenomics or metabolomics) into the screening procedure could also provide a more accurate investigation and prevent the drawbacks of the current methods. Moreover, a more accurate biomarkers investigation could be performed using a number of high-quality binary PPIs available in literature (Rolland et al., 2014) where a proteome-scale map of the human binary interactome is compared to alternative network maps in order to give a deeper insight into genotype-phenotype relationships. Finally, it will be necessary to develop an user-friendly interface to turn this methodological framework into a practical tool.

## Author contributions

AI and AO are joint first authors and both authors contributed equally. AI and AO prepared the computational codes and carried out all of the statistical analysis. CA, ID, and PL initiated and coordinated the work, guided the study design, supervised all data curation and analysis, and finalized all study conclusion. CA, ID, and PL are equal contributors. All the authors wrote, reviewed and approved the final manuscript.

### Conflict of interest statement

The authors declare that the research was conducted in the absence of any commercial or financial relationships that could be construed as a potential conflict of interest.

## References

[B1] Bonome T. Levine D. A. Shih J. Randonovich M. Pise-Masison C. A. Bogomolniy F.. (2008). A gene signature predicting for survival in suboptimally debulked patients with ovarian cancer. Cancer Res. 68, 5478–5486. 10.1158/0008-5472.CAN-07-6595 18593951 PMC7039050

[B2] Callagy G. M. Webber M. J. Pharoah P. D. Caldas C. (2008). Meta-analysis confirms BCL2 is an independent prognostic marker in breast cancer. BMC Cancer 8:153. 10.1186/1471-2407-8-153 18510726 PMC2430210

[B3] Candes E. Tao T. (2007). The Dantzig selector: statistical estimation when *p* is much larger than *n*. Ann. Stat. 35, 2313–2351. 10.1214/009053606000001523

[B4] Cox D. R. (1972). Regression models and life-tables. J. R. Stat. Soc. B Methodol. 187–220. 12343718

[B5] Cristiano B. E. Chan J. C. Hannan K. M. Lundie N. A. Marmy-Conus N. J. Campbell I. G.. (2006). A specific role for AKT3 in the genesis of ovarian cancer through modulation of G2-M phase transition. Cancer Res. 66, 11718–11725. 10.1158/0008-5472.CAN-06-1968 17178867

[B6] Desmedt C. Piette F. Loi S. Wang Y. Lallemand F. Haibe-Kains B.. (2007). Strong time dependence of the 76-gene prognostic signature for node-negative breast cancer patients in the transbig multicenter independent validation series. Clin. Cancer Res. 13, 3207–3214. 10.1158/1078-0432.CCR-06-2765 17545524

[B7] Dinulescu D. M. Ince T. A. Quade B. J. Shafer S. A. Crowley D. Jacks T. (2005). Role of K-ras and pten in the development of mouse models of endometriosis and endometrioid ovarian cancer. Nat. Med. 11, 63–70. 10.1038/nm1173 15619626

[B8] Dong Y. Walsh M. D. McGuckin M. A. Cummings M. C. Gabrielli B. G. Wright G. R.. (1997). Reduced expression of retinoblastoma gene product (pRB) and high expression of p53 are associated with poor prognosis in ovarian cancer. Int. J. Cancer, 74, 407–415. 10.1002/(SICI)1097-0215(19970822)74:4<407::AID-IJC8>3.0.CO;2-Z 9291430

[B9] Engler D. Li Y. (2009). Survival analysis with high-dimensional covariates: an application in microarray studies. Stat. Appl. Genet. Mol. Biol. 8, 1–22. 10.2202/1544-6115.1423 19222381 PMC2867485

[B10] Fan J. Feng Y. Wu Y. (2010). High-dimensional variable selection for cox's proportional hazards model, in Borrowing Strength: Theory Powering Applications–A Festschrift for Lawrence D. Brown, eds Berger J. O. Cai T. T. Johnstone I. M. (Beachwood, OH: Institute of Mathematical Statistics), 70–86.

[B11] Fan J. Li R. (2001). Variable selection via nonconcave penalized likelihood and its oracle properties. J. Am. Stat. Assoc. 96, 1348–1360. 10.1198/016214501753382273

[B12] Fan J. Lv J. (2008). Sure independence screening for ultrahigh dimensional feature space. J. R. Stat. Soc. B Stat. Methodol. 70, 849–911. 10.1111/j.1467-9868.2008.00674.x PMC2709408 19603084

[B13] Flesken-Nikitin A. Choi K.-C. Eng J. P. Shmidt E. N. Nikitin A. Y. (2003). Induction of carcinogenesis by concurrent inactivation of p53 and Rb1 in the mouse ovarian surface epithelium. Cancer Res. 63, 3459–3463. 12839925

[B14] Fröhlich H. (2014). Including network knowledge into Cox regression models for biomarker signature discovery. Biom. J. 56, 287–306. 10.1002/bimj.201300035 24430933

[B15] Gayther S. A. Mangion J. Russell P. Seal S. Barfoot R. Ponder B. A.. (1997). Variation of risks of breast and ovarian cancer associated with different germline mutations of the BRCA2 gene. Nat. Genet. 15, 103–105. 10.1038/ng0197-103 8988179

[B16] Gentleman R. C. Carey V. J. Bates D. M. Bolstad B. Dettling M. Dudoit S.. (2004). Bioconductor: open software development for computational biology and bioinformatics. Genome Biol. 5:R80. 10.1186/gb-2004-5-10-r80 15461798 PMC545600

[B17] Gong H. Wu T. T. Clarke E. M. (2014). Pathway-gene identification for pancreatic cancer survival via doubly regularized Cox regression. BMC Syst. Biol. 8(Suppl. 1):S3. 10.1186/1752-0509-8-s1-s3 24565114 PMC4080266

[B18] Gsur A. Preyer M. Haidinger G. Schatzl G. Madersbacher S. Marberger M.. (2002). A polymorphism in the UDP-glucuronosyltransferase 2B15 gene (D^85^Y) is not associated with prostate cancer risk. Cancer Epidemiol. Biomarkers Prev. 11, 497–498. 12010866

[B19] Gui J. Li H. (2005). Penalized Cox regression analysis in the high-dimensional and low-sample size settings, with applications to microarray gene expression data. Bioinformatics 21, 3001–3008. 10.1093/bioinformatics/bti422 15814556

[B20] Hudis C. A. (2007). Trastuzumabmechanism of action and use in clinical practice. N. Engl. J. Med. 357, 39–51. 10.1056/NEJMra043186 17611206

[B21] Hunter D. J. Kraft P. Jacobs K. B. Cox D. G. Yeager M. Hankinson S. E.. (2007). A genome-wide association study identifies alleles in FGFR2 associated with risk of sporadic postmenopausal breast cancer. Nat. Genet. 39, 870–874. 10.1038/ng2075 17529973 PMC3493132

[B22] Huttenhower C. Haley E. M. Hibbs M. A. Dumeaux V. Barrett D. R. Coller H. A.. (2009). Exploring the human genome with functional maps. Genome Res. 19, 1093–1106. 10.1101/gr.082214.108 19246570 PMC2694471

[B23] Iuliano A. Occhipinti A. Angelini C. De Feis I. Lió P. (2014). Applications of network-based survival analysis methods for pathways detection in cancer, in Computational Intelligence Methods for Bioinformatics and Biostatistics, eds Di Serio C. Liò P. Nonis A. Tagliaferri R. (Springer), 76–88.

[B24] Jeong H.-H. Kim S. Y. Wee K. Sohn K.-A. (2015). Investigating the utility of clinical outcome-guided mutual information network in network-based Cox regression. BMC Syst. Biol. 9:1. 10.1186/1752-0509-9-S1-S8 25708115 PMC4331683

[B25] Kao K.-J. Chang K.-M. Hsu H.-C. Huang A. T. (2011). Correlation of microarray-based breast cancer molecular subtypes and clinical outcomes: implications for treatment optimization. BMC Cancer 11:143. 10.1186/1471-2407-11-143 21501481 PMC3094326

[B26] Karapetis C. S. Khambata-Ford S. Jonker D. J. O'Callaghan C. J. Tu D. Tebbutt N. C.. (2008). K-ras mutations and benefit from cetuximab in advanced colorectal cancer. N. Engl. J. Med. 359, 1757–1765. 10.1056/NEJMoa0804385 18946061

[B27] Katoh M. (2005). WNT/PCP signaling pathway and human cancer (review). Oncol. Rep. 14, 1583–1588. 10.3892/or.14.6.1583 16273260

[B28] Kearns M. Ron D. (1999). Algorithmic stability and sanity-check bounds for leave-one-out cross-validation. Neural Comput. 11, 1427–1453. 10.1162/089976699300016304 10423502

[B29] Kikuchi R. Tsuda H. Kanai Y. Kasamatsu T. Sengoku K. Hirohashi S.. (2007). Promoter hypermethylation contributes to frequent inactivation of a putative conditional tumor suppressor gene connective tissue growth factor in ovarian cancer. Cancer Res. 67, 7095–7105. 10.1158/0008-5472.CAN-06-4567 17671176

[B30] Kohavi R. (1995). A study of cross-validation and bootstrap for accuracy estimation and model selection, in IJCAI, Vol. 14 (Stanford, CA), 1137–1145.

[B31] Li C. Li H. (2008). Network-constrained regularization and variable selection for analysis of genomic data. Bioinformatics 24, 1175–1182. 10.1093/bioinformatics/btn081 18310618

[B32] Li C. Li H. (2010). Variable selection and regression analysis for graph-structured covariates with an application to genomics. Ann. Appl. Stat. 4, 1498. 10.1214/10-AOAS332 22916087 PMC3423227

[B33] Liby T. A. Spyropoulos P. Buff Lindner H. Eldridge J. Beeson C. Hsu T.. (2012). Akt3 controls vascular endothelial growth factor secretion and angiogenesis in ovarian cancer cells. Int. J. Cancer 130, 532–543. 10.1002/ijc.26010 21351097 PMC3189303

[B34] Martinez-Ledesma E. Verhaak R. G. Treviño V. (2015). Identification of a multi-cancer gene expression biomarker for cancer clinical outcomes using a network-based algorithm. Sci. Rep. 5:11966. 10.1038/srep11966 26202601 PMC5378879

[B35] Mattie M. D. Benz C. C. Bowers J. Sensinger K. Wong L. Scott G. K.. (2006). Optimized high-throughput microrna expression profiling provides novel biomarker assessment of clinical prostate and breast cancer biopsies. Mol. Cancer 5:24. 10.1186/1476-4598-5-24 16784538 PMC1563474

[B36] Molinaro A. M. Simon R. Pfeiffer R. M. (2005). Prediction error estimation: a comparison of resampling methods. Bioinformatics 21, 3301–3307. 10.1093/bioinformatics/bti499 15905277

[B37] Nakatani K. Thompson D. A. Barthel A. Sakaue H. Liu W. Weigel R. J.. (1999). Up-regulation of Akt3 in estrogen receptor-deficient breast cancers and androgen-independent prostate cancer lines. J. Biol. Chem. 274, 21528–21532. 10.1074/jbc.274.31.21528 10419456

[B38] Naora H. Montell D. J. (2005). Ovarian cancer metastasis: integrating insights from disparate model organisms. Nat. Rev. Cancer 5, 355–366. 10.1038/nrc1611 15864277

[B39] Raghupathi W. Raghupathi V. (2014). Big data analytics in healthcare: promise and potential. Health Inf. Sci. Syst. 2:3. 10.1186/2047-2501-2-3 25825667 PMC4341817

[B40] Refaeilzadeh P. Tang L. Liu H. (2009). Cross-validation, in Encyclopedia of Database Systems, eds Liu L. Özsu M. T. (New York, NY: Springer), 532–538. 10.1007/978-0-387-39940-9_565

[B41] Rikova K. Guo A. Zeng Q. Possemato A. Yu J. Haack H.. (2007). Global survey of phosphotyrosine signaling identifies oncogenic kinases in lung cancer. Cell 131, 1190–1203. 10.1016/j.cell.2007.11.025 18083107

[B42] Rolland T. Taşan M. Charloteaux B. Pevzner S. J. Zhong Q. Sahni N.. (2014). A proteome-scale map of the human interactome network. Cell 159, 1212–1226. 10.1016/j.cell.2014.10.050 25416956 PMC4266588

[B43] Schuijer M. Berns E. M. (2003). TP53 and ovarian cancer. Hum. Mutat. 21, 285–291. 10.1002/humu.10181 12619114

[B44] Simon N. Friedman J. Hastie T. Tibshirani R. (2011a). Regularization paths for Cox's proportional hazards model via coordinate descent. J. stat. Softw. 39, 1–13. 10.18637/jss.v039.i05 27065756 PMC4824408

[B45] Simon R. M. Subramanian J. Li M.-C. Menezes S. (2011b). Using cross-validation to evaluate predictive accuracy of survival risk classifiers based on high-dimensional data. Brief. Bioinform. 12, 203–214. 10.1093/bib/bbr001 21324971 PMC3105299

[B46] Sun H. Lin W. Feng R. Li H. (2014). Network-regularized high-dimensional Cox regression for analysis of genomic data. Stat. Sin. 24:1433. 10.5705/ss.2012.317 26316678 PMC4549005

[B47] Tapper J. Kettunen E. El-Rifai W. Seppälä M. Andersson L. C. Knuutila S. (2001). Changes in gene expression during progression of ovarian carcinoma. Cancer Genet. Cytogenet. 128, 1–6. 10.1016/S0165-4608(01)00386-7 11454421

[B48] The Cancer Genome Atlas Research Network (2011). Integrated genomic analyses of ovarian carcinoma. Nature 474, 609–615. 10.1038/nature10166 21720365 PMC3163504

[B49] Thomadaki H. Talieri M. Scorilas A. (2007). Prognostic value of the apoptosis related genes BCL2 and BCL2L12 in breast cancer. Cancer Lett. 247, 48–55. 10.1016/j.canlet.2006.03.016 16647810

[B50] Tibshirani R. (1996). Regression shrinkage and selection via the lasso. J. R. Stat. Soc. B Methodol. 267–288. 10.1002/(SICI)1097-0258(19970228)16:4<385::AID-SIM380>3.0.CO;2-326059498

[B51] Tibshirani R. (1997). The Lasso method for variable selection in the cox model. Stat. Med. 16, 385–395. 9044528 10.1002/(sici)1097-0258(19970228)16:4<385::aid-sim380>3.0.co;2-3

[B52] van Houwelingen H. C. Bruinsma T. Hart A. A. van't Veer L. J. Wessels L. F. (2006). Cross-validated Cox regression on microarray gene expression data. Stat. Med. 25, 3201–3216. 10.1002/sim.2353 16143967

[B53] Vasselli J. R. Shih J. H. Iyengar S. R. Maranchie J. Riss J. Worrell R.. (2003). Predicting survival in patients with metastatic kidney cancer by gene-expression profiling in the primary tumor. Proc. Natl. Acad. Sci. U.S.A. 100, 6958–6963. 10.1073/pnas.1131754100 12777628 PMC165812

[B54] Wang B. Mezlini A. M. Demir F. Fiume M. Tu Z. Brudno M.. (2014). Similarity network fusion for aggregating data types on a genomic scale. Nat. Methods 11, 333–337. 10.1038/nmeth.2810 24464287

[B55] Wegman P. Elingarami S. Carstensen J. Stal O. Nordenskjold B. Wingren S. (2007). Genetic variants of CYP3A5, CYP2D6, SULT1A1, UGT2B15 and tamoxifen response in postmenopausal patients with breast cancer. Breast Cancer Res. 9:R7. 10.1186/bcr1640 17244352 PMC1851378

[B56] Wooster R. Bignell G. Lancaster J. Swift S. Seal S. Mangion J.. (1995). Identification of the breast cancer susceptibility gene BRCA2. Nature 378, 789–792. 10.1038/378789a0 8524414

[B57] Wu T. T. Wang S. (2013). Doubly regularized Cox regression for high-dimensional survival data with group structures. Stat. Interface 6, 175–186. 10.4310/SII.2013.v6.n2.a2

[B58] Wu Y. (2012). Elastic net for Coxs proportional hazards model with a solution path algorithm. Stat. Sin. 22:27. 10.5705/ss.2010.107 23226932 PMC3515861

[B59] Yang G. Truong L. D. Timme T. L. Ren C. Wheeler T. M. Park S. H.. (1998). Elevated expression of caveolin is associated with prostate and breast cancer. Clin. Cancer Res. 4, 1873–1880. 9717814

[B60] Yang Y. Zou H. (2012). A cocktail algorithm for solving the elastic net penalized Coxs regression in high dimensions. Stat. Sin. 6, 167–173.

[B61] Zhang W. Ota T. Shridhar V. Chien J. Wu B. Kuang R. (2013). Network-based survival analysis reveals subnetwork signatures for predicting outcomes of ovarian cancer treatment. PLoS Comput. Biol. 9:e1002975. 10.1371/journal.pcbi.1002975 23555212 PMC3605061

[B62] Zou H. (2006). The adaptive lasso and its oracle properties. J. Am. Stat. Assoc. 101, 1418–1429. 10.1198/016214506000000735

[B63] Zou H. Hastie T. (2005). Regularization and variable selection via the elastic net. J. R. Stat. Soc. B Methodol. 67, 301–320. 10.1111/j.1467-9868.2005.00503.x

