# Supplementary figures and images for: Cancer Markers Selection Using Network-Based Cox Regression: A Methodological and Computational Practice
Source: Front Physiol. 2016 Jun 17;7:208. doi: 10.3389/fphys.2016.00208 (PMC4911360; doi:10.3389/fphys.2016.00208)

AdaLnet (Not-Overlapped Pathways)

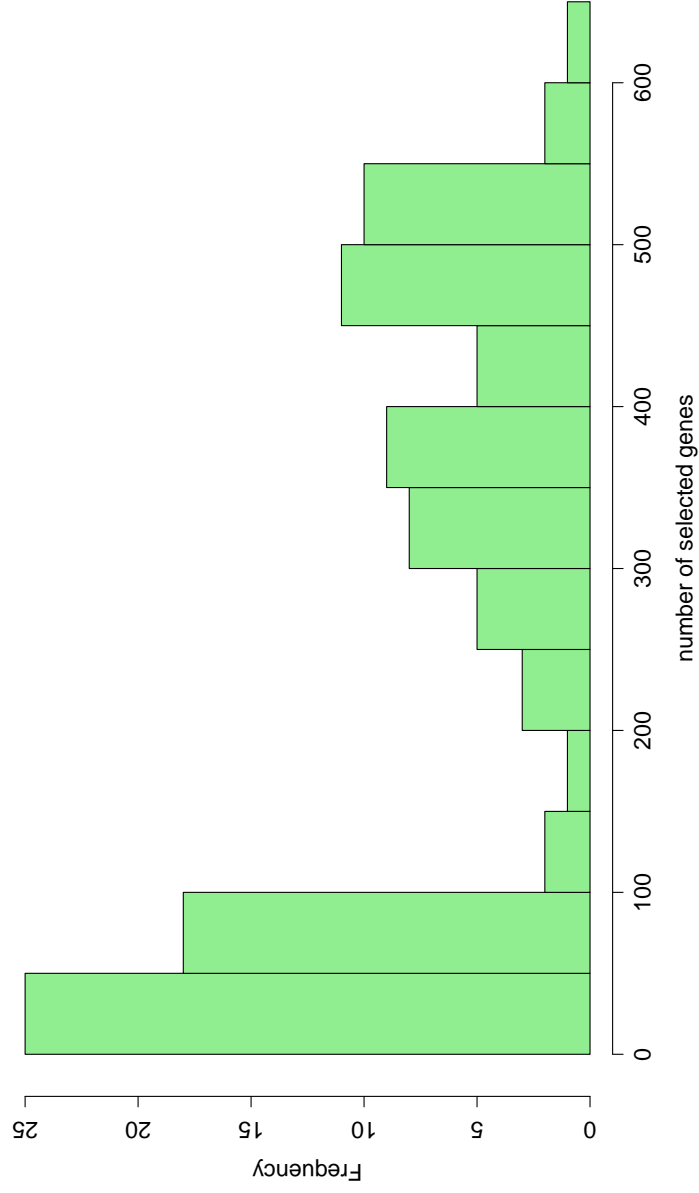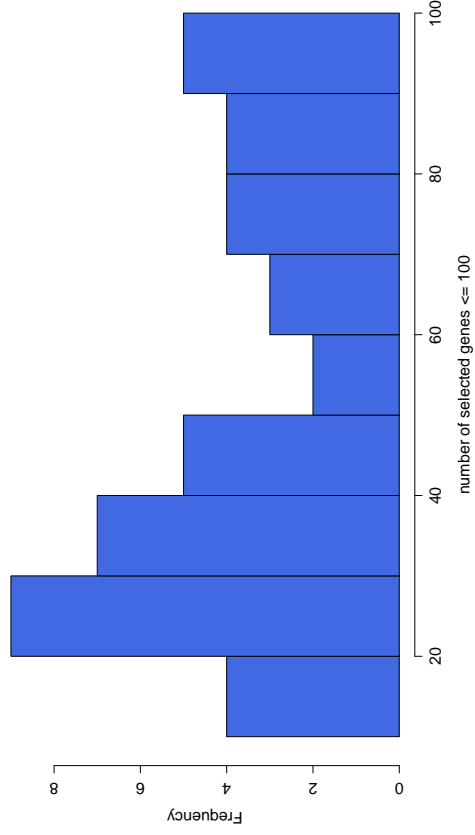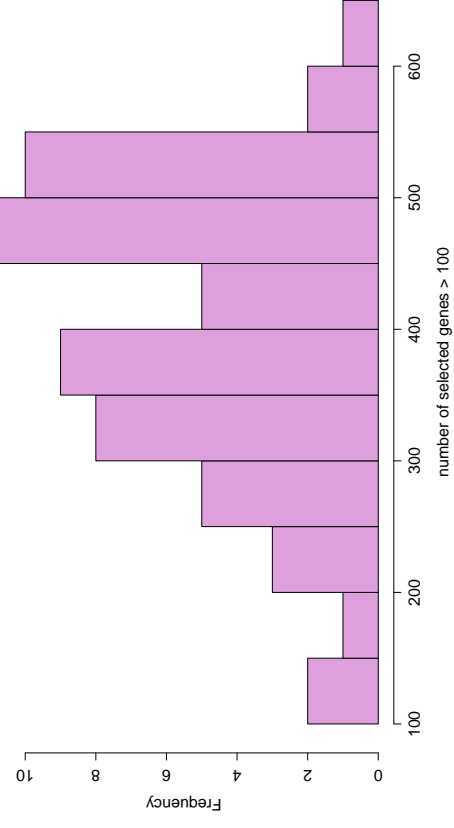

fastcox (Not-Overlapped Pathways)

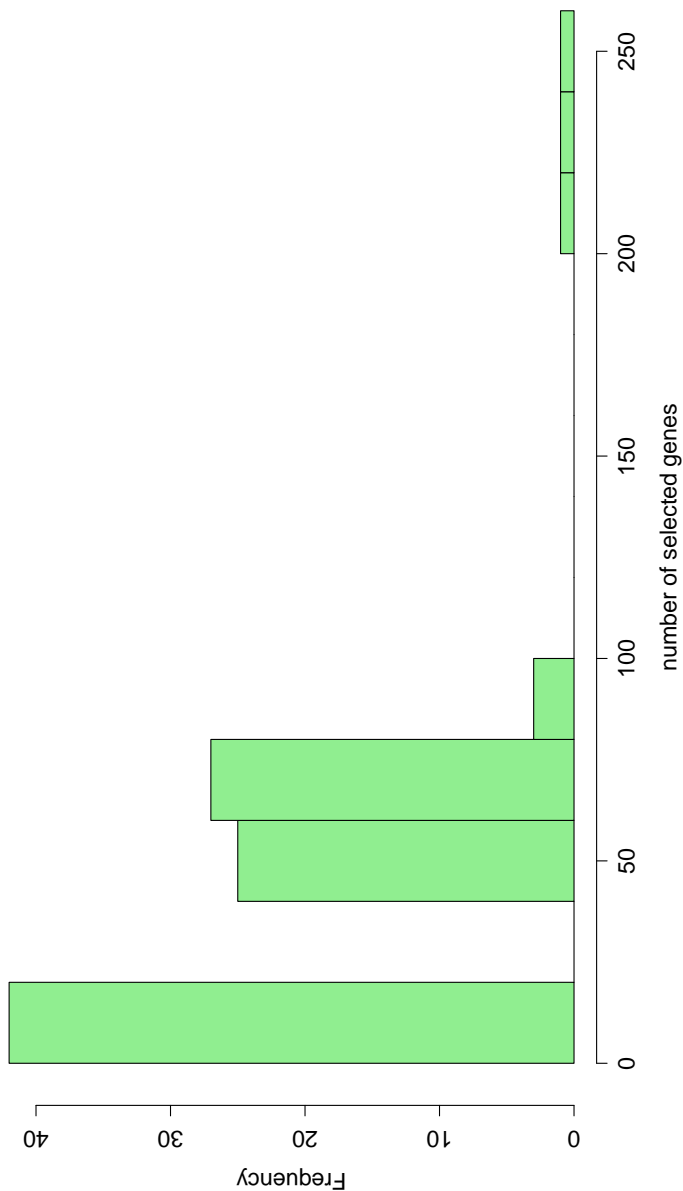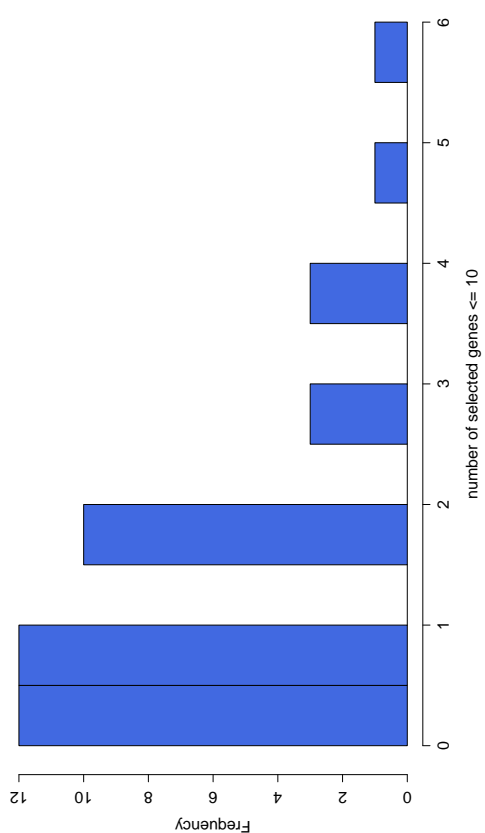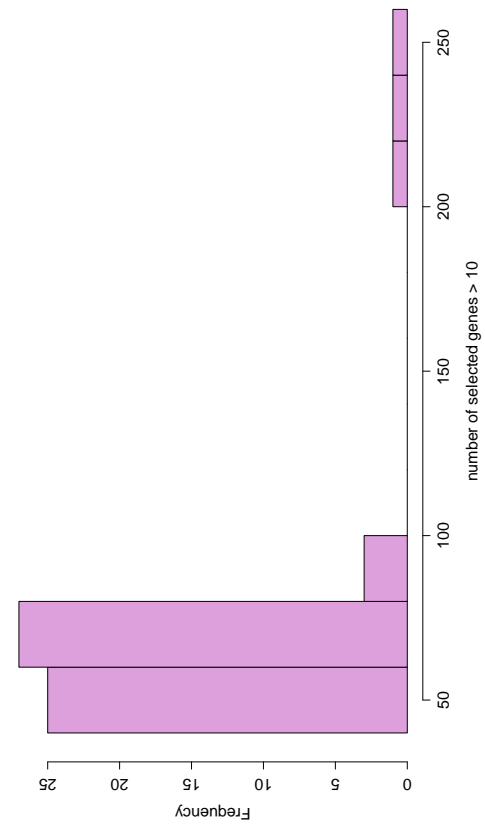

Supplement: Supplementary file 3 [file Image1.PDF]

AdaLnet (Overlapped Pathways)

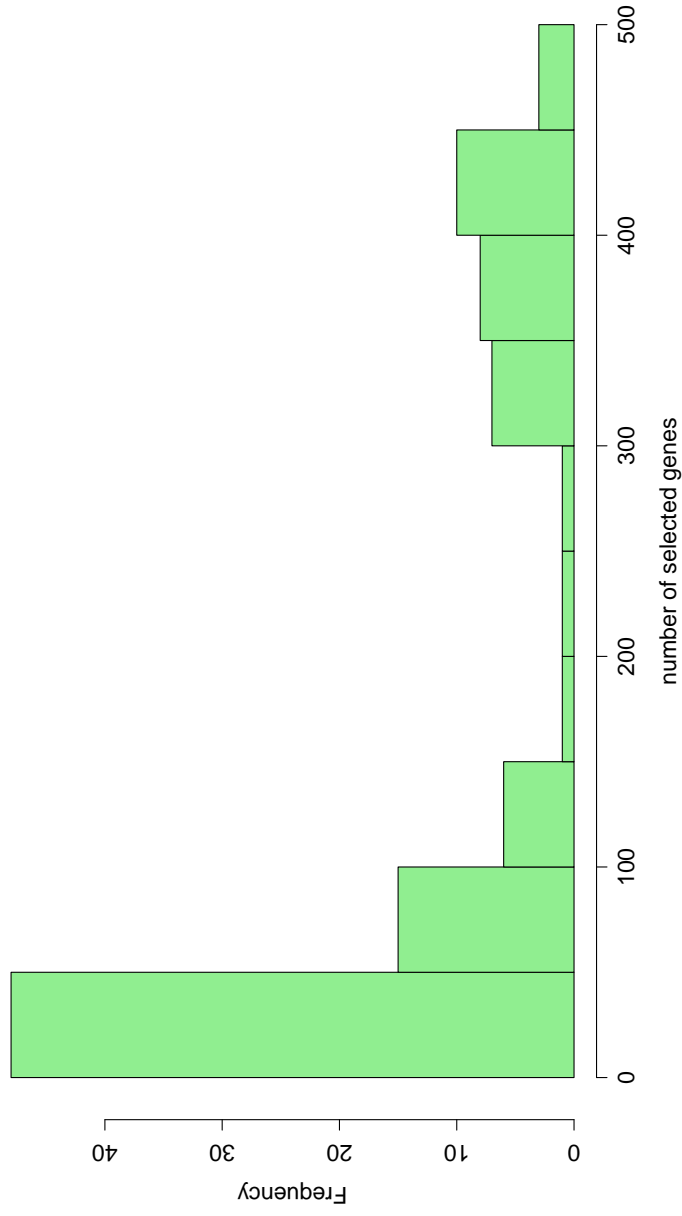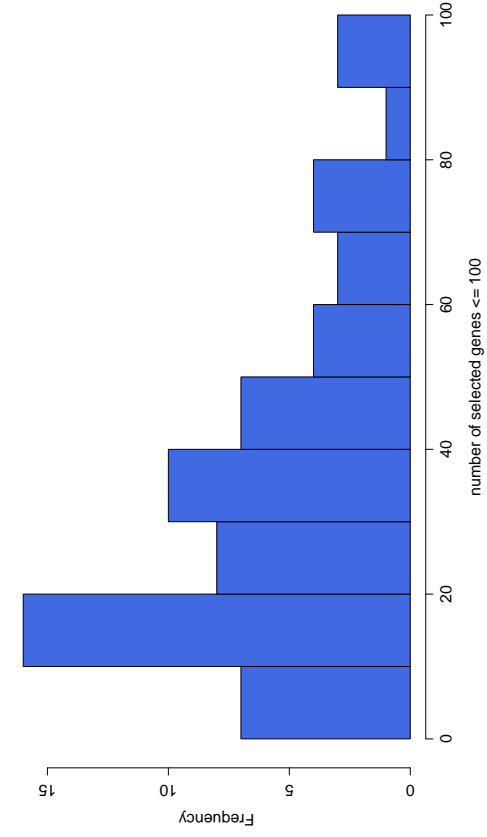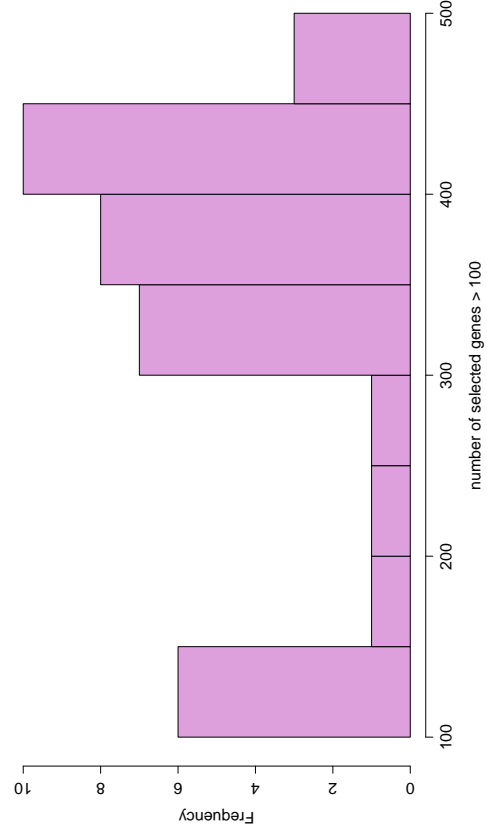

fastcox (Overlapped Pathways)

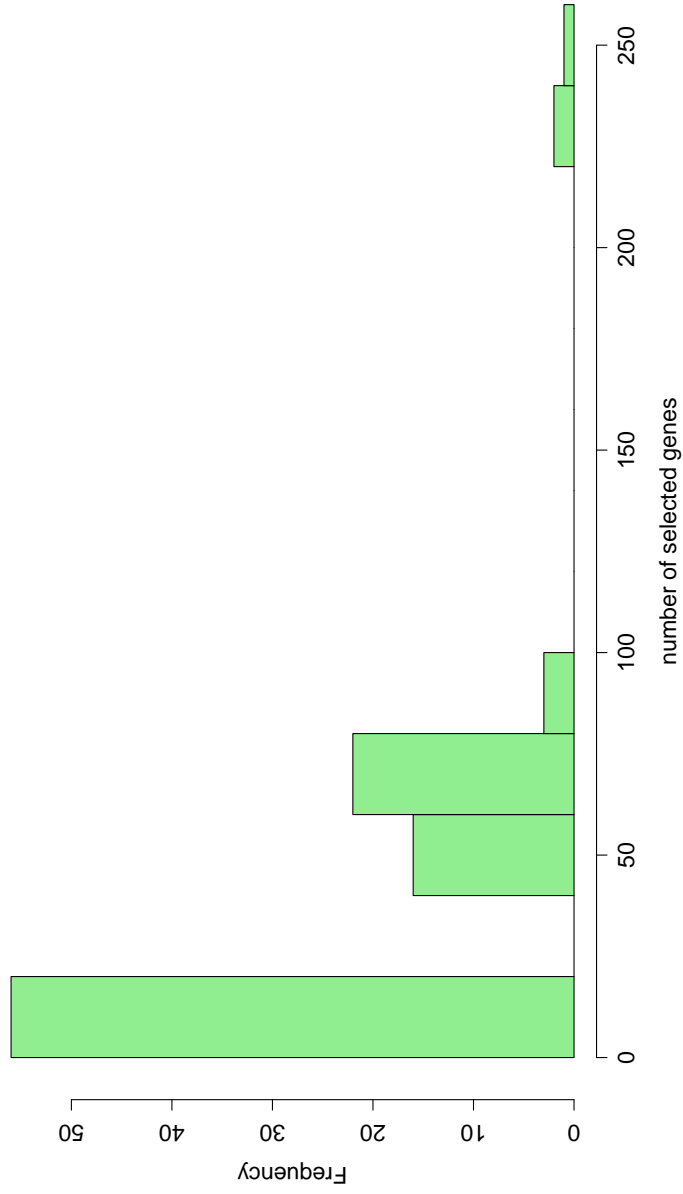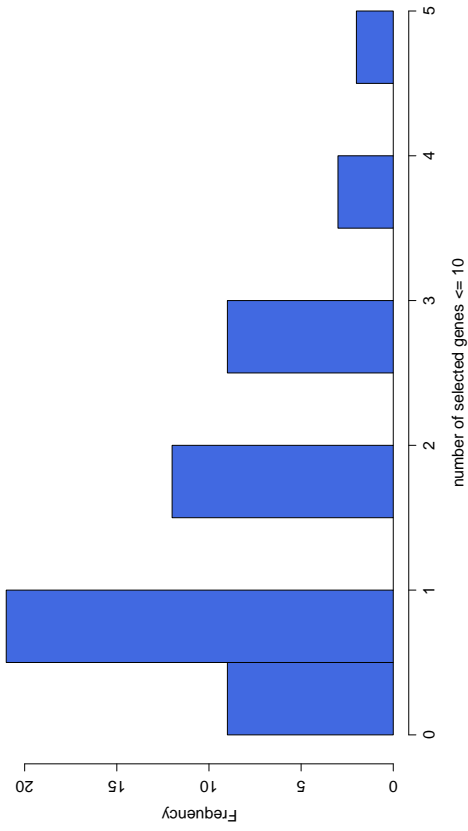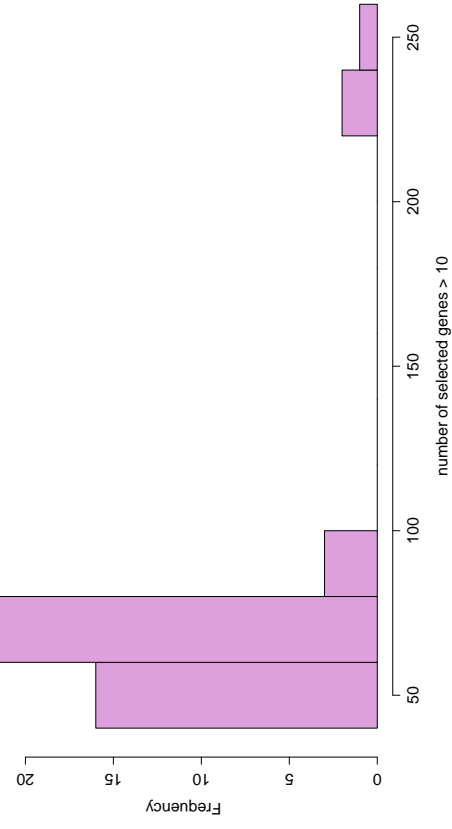

Supplement: Supplementary file 4 [file Image2.PDF]
